# Double-jeopardy: scRNA-seq doublet/multiplet detection using multi-omic profiling

**DOI:** 10.1016/j.crmeth.2021.100008

**Published:** 2021-05-24

**Authors:** Bo Sun, Emmanuel Bugarin-Estrada, Lauren Elizabeth Overend, Catherine Elizabeth Walker, Felicia Anna Tucci, Rachael Jennifer Mary Bashford-Rogers

**Affiliations:** 1 Wellcome Centre for Human Genetics, University of Oxford, Oxford, UK; 2 Oxford Autoimmune Neurology Group, Nuffield Department of Clinical Neurosciences, University of Oxford, Oxford, UK

**Keywords:** single-cell transcriptomics, B cell receptor, T cell receptor, doublets, multi-omics profiling, ADT, CITE-seq

## Abstract

The computational detection and exclusion of cellular doublets and/or multiplets is a cornerstone for the identification the true biological signals from single-cell RNA sequencing (scRNA-seq) data. Current methods do not sensitively identify both heterotypic and homotypic doublets and/or multiplets. Here, we describe a machine learning approach for doublet/multiplet detection utilizing VDJ-seq and/or CITE-seq data to predict their presence based on transcriptional features associated with identified hybrid droplets. This approach highlights the utility of leveraging multi-omic single-cell information for the generation of high-quality datasets. Our method has high sensitivity and specificity in inflammatory-cell-dominant scRNA-seq samples, thus presenting a powerful approach to ensuring high-quality scRNA-seq data.

## Highlights

• Doublets and multiplets in single-cell RNA-seq confound biological results
• Single-cell VDJ-seq and CITE-seq can aid the identification of doublets/multiplets
• Machine learning can identify further heterotypic and homotypic doublets/multiplets

## Motivation

Single-cell RNA sequencing (scRNA-seq) techniques are transforming our understanding of multicellular organisms, disease states, and cellular heterogeneity. The aggregation of two or more cells into single droplets (doublets/multiplets) during the cell capture step of scRNA-seq resulting in hybrid transcriptomes can lead to false discoveries of rare cell types, intermediate cell states, and disease-associated transcriptomic signatures. Current methods do not sensitively identify many doublets/multiplets. Here, we address this doublet/multiplet detection issue utilizing VDJ-seq and/or CITE-seq, and apply machine learning to predict their presence based on transcriptional features associated with identified hybrid droplets, and ultimately scRNA-seq data quality.

## Graphical abstract

**Figure:**
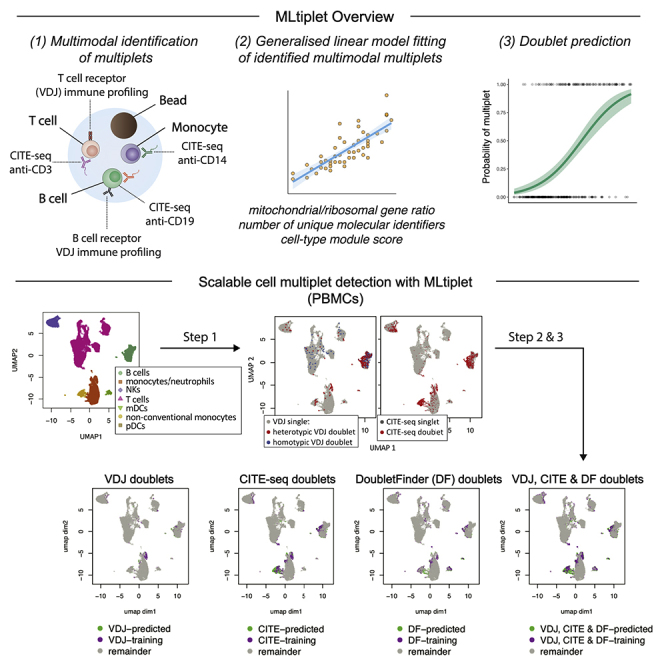

## Teaser

Sun et al. demonstrate that doublets/multiplets might be identified from single-cell RNA-seq datasets through analysis of mutually exclusive VDJ-seq and/or CITE-seq features. Moreover, we use machine learning approaches for doublet/multiplet detection utilizing VDJ-seq and/or CITE-seq information to predict their presence based on transcriptional features associated with identified hybrid droplets.

## Introduction

The use of single-cell RNA sequencing (scRNA-seq) techniques has revolutionized the characterization of complex biological systems in health and diseased states; however, its fundamental success relies on the true representations of high-quality single cells. Droplet-based scRNA-seq technologies have allowed for the capture and analysis of 1,000s–100,000s of cells in single experiments at reduced per-cell cost. The success of single-cell experiments depends on the accurate capture of a single cell from a prepared cell suspension. However, many scRNA-seq techniques face significant limitations, including the capture of doublets (or multiplets) and/or low-quality or dying cells, which potentially confounds biological results. Doublets and multiplets are defined as the aggregation of two or more cells into single droplets during the cell capture step of scRNA-seq, resulting in hybrid transcriptomes (Zheng et al., 2017; Wolock et al., 2019; McGinnis et al., 2019). Hybrid transcriptomes might be derived from the aggregation of two or more intact cells and/or dying and/or broken cells and can be homotypic (comprised of cells that are transcriptionally similar) or heterotypic (comprised of cells with dissimilar gene expression). Conditions resulting in a propensity for cellular clumping might contribute to the formation of cellular doublets or the non-specific binding of detection antibodies. The aggregation of cells undergoing apoptosis is well described in flow cytometry experiments (Cui et al., 2016; Kuonen et al., 2010). Although most single-cell experiments perform dead cell exclusion, the protocols can be protracted and result in further cell death downstream. These processes can result in false discoveries of rare cell types, intermediate cell states and disease-associated transcriptomic signatures (Stegle et al., 2015; Ilicic et al., 2016). Although prospective experimental planning might reduce the frequency of doublet/multiplet occurrence, such as capturing fewer cells during scRNA-seq library preparation, doublet and/or multiplet capture is a problem in all experimental setups (Zheng et al., 2017; Wolock et al., 2019; McGinnis et al., 2019).

The computational detection and exclusion of doublet or multiplet artifacts is required to accurately analyze the true biological signals in single-cell data. Doublet detection might be achieved experimentally through cell hashing (Stoeckius et al., 2018) (pooling of multiple samples labeled with distinct oligo-tagged antibodies against ubiquitously expressed surface proteins) or through genotype-based multiplexing (Kang et al., 2018) (genetic variation between multiplexed sample donors to determine the sample identity of each cell and detect droplets containing two or more cells); however, these data modalities are not suitable or included in many scRNA-seq experiments. Current methods for identification of same-sample doublets and/or multiplets include DoubletFinder (McGinnis et al., 2019), Scrublet (Wolock et al., 2019), DoubletDecon (Depasquale et al., 2019), and scds (Bais and Kostka, 2020), all of which primarily identify only heterotypic doublets, and some methods require the prior estimation of multiplet rate, which is not possible to evaluate (McGinnis et al., 2019). There are no robust identification methods for both heterotypic and homotypic doublets and/or multiplets.

Here, we describe approaches for the detection of both heterotypic and homotypic doublets and/or multiplets. This is possible through the use of recent multi-omic approaches in scRNA-seq experiments that characterize the proteomic (Stoeckius et al., 2017) and immune receptor profiles of single cells (Azizi et al., 2018) offering further modalities of multiplet identification that build upon current gene expression-based tools (Wolock et al., 2019; McGinnis et al., 2019). The use of barcoded oligonucleotide-conjugated antibodies that target cell-surface markers (CITE-seq) can potentially identify cell multiplets through the occurrence of co-staining for cell-type-specific canonical markers, such as CD3 and CD19, exclusive markers for T and B cells, respectively. Similarly, the expression of more than one clonally distinct T or B cell receptor (TCR or BCR, respectively) chain in a single T or B cell is a rare occurrence. In B cells, double light chains occur at 2%–10% in murine models (Casellas et al., 2007), whereas allelically inclusive expression of double heavy chains is incredibly rare at reported frequencies of 0.01% (Barreto and Cumano, 2000). In T cells, the expression of more than one distinct alpha chain (TCRα) has been estimated to occur in 1%–10% of human peripheral T cells (Padovan et al., 1993), whereas double beta chain (TCRβ) are rarer at frequencies of <1%. The possibility of a T or B cell endogenously expressing the respective cell-type-specific receptor (Ahmed et al., 2019) remains controversial and has yet to be reproduced. Therefore, droplets resembling a mixture of B and T cell CITE-seq or VDJ-seq profiles might be enriched in doublets and/or multiplets.

We therefore propose a computational cell doublet/multiplet detection approach, MLtiplet, that leverages the additional granularity of multimodal scRNA-seq experiments using CITE-seq and immune receptor profiling, respectively. This approach identified droplets that correspond to mixed-cell droplet profiles, including those with mutually exclusive CITE-seq profiles or multiple TCR/BCR profiles. As these CITE-seq or TCR/BCR profiles only identify a subset of *possible doublets and/or multiplets*, we subsequently applied a generalized linear model to fit the profile of these *true doublets and/or multiplets* compared with the remainder of the droplets. The model was then used as a classifier to detect doublets and/or multiplets for which the CITE-seq or VDJ-seq data were not available or appropriate. This approach incorporates features of the transcriptomic profiles into a single model that statistically distinguishes doublets and/or multiplets from true singlets. These transcriptional features include the relative number of mRNA molecules (nUMIs), and apoptosis-associated gene signatures. We show that doublets and/or multiplets identified from both CITE-seq and VDJ-seq data might be used as a combined training dataset for MLtiplet for highest sensitivity and specificity.

## Results

**Figure 1:**
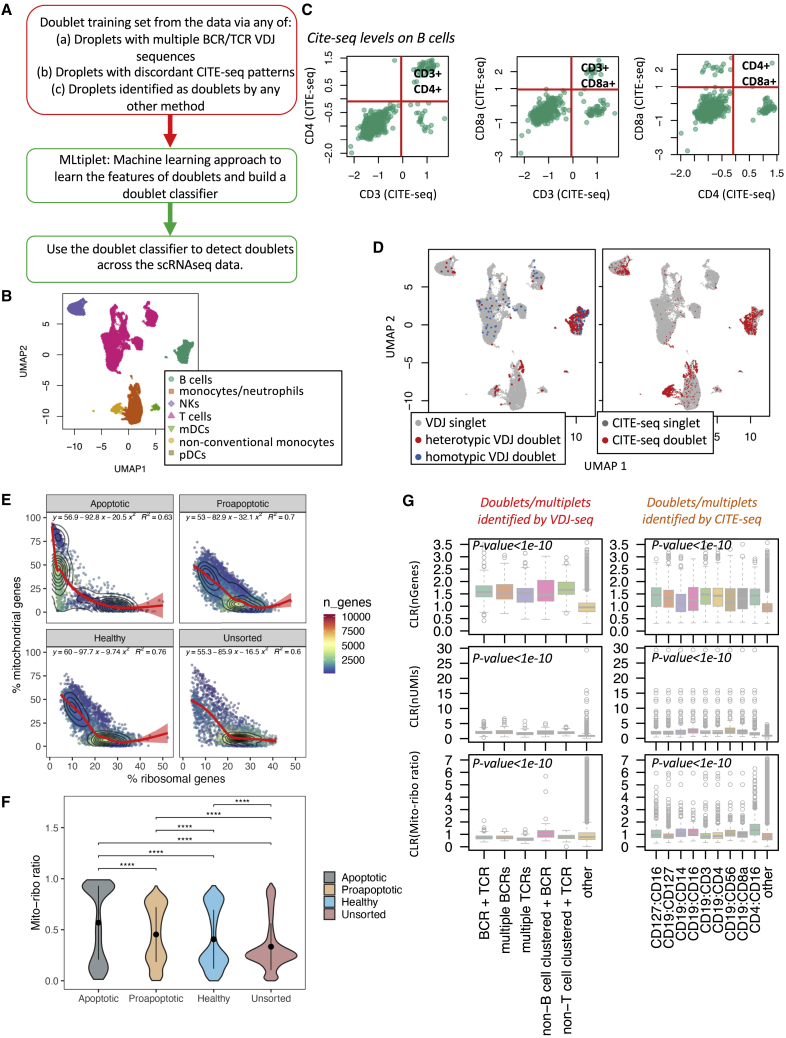
Multi-omics aids the identification of doublets and/or multiplets (A) Schematic of approach to identify scRNA-seq doublets and/or multiplets using the CITE-seq and VDJ modalities. Droplets with a transcriptome resembling non-B or non-T cells that captured BCR or TCR sequences, respectively, were considered as potential doublets and/or multiplets. (B) Uniform manifold approximation and projection (UMAP) dimensionality reduction of three healthy PBMC datasets colored by cell type. (C) Examples of the CITE-seq levels between CD3, CD4, and CD8 for the three individuals for the B cell cluster, with the red lines corresponding to the CITE-seq positivity thresholds. (D) UMAP dimensionality reduction of three healthy PBMCs colored by VDJ doublets (left, co-capture of discordant VDJs) or CITE-seq doublets (right, co-capture of the corresponding mutually exclusive CITE-seq pair). Homotypic doublets were defined as those containing multiple BCRs or multiple TCRs, and the heterotypic doublets are defined as the remainder (droplets containing both BCR(s) and TCR(s) or droplets containing BCRs or TCRs that do not have transcriptional profiles that resemble B or T cells, respectively). (E) Generalized additive models fitted on percentage mitochondrial genes versus percentage ribosomal genes, R^2^ values shown top right; from the HEK293 dataset. (F) Mito-ribo ratio values per enriched HEK293 populations. p values were calculated across groups by using Wilcoxon test with Bonferroni correction for pairwise comparisons. (G) The relative numbers of genes (nGenes), number of RNA molecules (nUMI), mito-ribo ratio (mitoribo_ratio), and nUMIs_VDJ per droplet for the VDJ-identified doublets and/or multiplets (left) and the CITE-seq identified doublets and/or multiplets (right). “Other” refers to droplets that were not identified as doublets and/or multiplets from the VDJ-seq or CITE-seq data. The p values of the differences between the feature distributions of the doublet/multiplets detected and the remainder of the droplets provided (two-sided Wilcoxon test). ∗∗∗∗p < 0.00005 (Wilcoxon test). Abbreviation is as follows: CLR, centered log-ratio transformed.

Here, we propose a model-based classification of doublets based on the profiles of identified mixed-cell droplets (Figures 1A and S1). We applied these approaches to publicly available datasets containing RNA-seq, CITE-seq, and VDJ-seq modalities of peripheral blood mononuclear cells (PBMCs) from three healthy individuals (https://support.10xgenomics.com/single-cell-vdj/datasets). These were pre-processed according to the Seurat analysis pipeline to exclude low-quality droplets (with low numbers of captured genes and RNA molecules) and were batch corrected through *harmony* (see the STAR Methods). A total of 26,080 droplets were retained after filtering (7,024–11,382 per sample), for which the broad immune cell types were annotated through differential gene expression and CITE-seq marker expression (Figures 1B and S1C).

The first step for doublet/multiplet detection is to identify mixed-cell droplet profiles that can be deduced from the data. This might be achieved through the leveraging of CITE-seq and immune receptor profiling. The CITE-seq approach allows for the potential identification of cell multiplets through the occurrence of co-staining for cell-type-exclusive canonical markers that are not expected to be co-expressed. For example, the majority of B cells identified from the scRNA-seq gene expression do not have high CITE-seq levels for the T cell markers CD3, CD4, or CD8 (Figures 1C, S1D, and S1E). However, of the B cells that have high CITE-seq levels for these T cell markers, the CITE-seq patterns reflect those expected for hybrid of B cells and CD4^+^ or CD8^+^ T cells: high CITE-seq levels of CD3 and CD4 or CD3 and CD8 together, but not CD4 and CD8 together. This is suggestive of true doublets reflecting a mixture of CITE-seq patterns that might be used to infer doublets and/or multiplets. From 26,080 filtered cells after quality control, 2,068 droplets were identified with CITE-seq profiles resembling mixed-cell-type doublet/multiplet droplets (Table S1). Each cell-type-specific mutually exclusive CITE-seq pair is observed in different UMAP clusters (Figures 1D and S2A), with B-T cell hybrids (CD19^+^CD4^+^, CD19^+^CD8^+^, and CD19^+^CD3^+^ droplets) observed primarily in the “B cell” cluster, and B-cell-myeloid hybrids (CD19^+^CD16^+^) and T cell-neutrophil/monocyte hybrids (CD4^+^CD16^+^) observed primarily in the “neutrophils/monocyte” cluster.

Similarly, the VDJ-seq approach allows for the potential identification of doublets and/or multiplets through the occurrence of droplet co-capture of multiple functional BCR heavy or light chains (suggestive of two B cells captured), multiple functional TCR alpha or beta chains (suggestive of two T cells captured), or combinations of BCRs and TCRs (suggestive of a B cell and a T cell captured). In addition, droplets with a transcriptome resembling non-B or non-T cells that captured BCR or TCR sequences, respectively, were considered as potential doublets and/or multiplets. Given the common dual TCR alpha chain expression, these droplets were not included as markers of potential doublets and/or multiplets. From 26,080 filtered cells after QC, 835 droplets were identified with VDJ-seq profiles resembling doublet/multiplet droplets (Table S1), and are enriched in the B cell and T cell clusters (Figures 1D and S2B).

As these CITE-seq or TCR/BCR profiles will only identify a subset of *possible doublets and/or multiplets*, the second step is to then apply a model to fit the profile of these *identified doublets and/or multiplets* to be used as a classifier to predict doublets and/or multiplets in the remaining cells, such as for those where the CITE-seq or VDJ-seq was not available/appropriate. This approach might incorporate any single-cell cellular, transcriptional, CITE-seq, or VDJ-seq variable into a single model that statistically distinguishes doublets and/or multiplets from true singlets, such as the relative number of captured genes and RNA molecules per droplet that are expected to be elevated in droplets that captured more than one cell.

We also consider a transcriptional marker for the identification of low-quality or dying cells in scRNA-seq data given the propensity of these cells to aggregate (Cui et al., 2016; Kuonen et al., 2010), thus potentially providing a useful marker for dying/low-quality cells that are more prone to forming doublet/multiplet aggregates. Current computational identification of low-quality cells relies on arbitrary thresholds of percentage representation of mitochondrial gene counts (Ilicic et al., 2016); however, such thresholds have been shown to perform poorly on ground truth datasets (Ordonez-Rueda et al., 2020). We hypothesized that downregulation of RNA encoding ribosomal proteins (rRNA), associated with cellular stress (Albert et al., 2019) and apoptosis (Lin et al., 1994), is a technical droplet feature that might improve sensitivity for detection of apoptotic or pre-apoptotic cells. Indeed, principal-component analysis on a ground truth scRNA-seq dataset of FACS sorted apoptotic HEK cells (Ordonez-Rueda et al., 2020) revealed that the main variables driving dead cell identification were low percentage ribosomal genes and high percentage mitochondrial genes; representing 49.7% of all variance explained by the first principle component (Figures 1E and S2C–S2E). Therefore, we created an additional variable to be included in the doublet detection model, named the mito-ribo ratio, calculated by the proportion of mitochondrial RNA (mtRNA) divided by the sum of the proportion of rRNA and mtRNA. Indeed, the mito-ribo ratio was elevated in apoptotic cells (Figure 1F). By thresholding on the local minimum (0.47) of a fitted Gaussian mixture model (GMM), we were able to discriminate 84% of late apoptotic cells with >1,537 detected genes, threshold for doublets identified by a GMM (traditional arbitrary filter is >2,500 genes), and observed a trend toward higher scores in early apoptotic cells (Figure 1F).

We next tested whether these features might discriminate the identified doublets and/or multiplets from the remainder of the droplets (which should be enriched for singlets). As expected, the relative number of genes (nGenes) and mRNA molecules (nUMIs) detected was significantly higher in the identified doublets and/or multiplets through the VDJ-seq and CITE-seq approaches than in the remainder of droplets (p < 1e−10, Figure 1G). The mito-ribo ratio varied significantly between droplet types and by cell cycle (Figure S2F) (p < 1e−10, ANCOVA using sample as an additional covariate), which was consistent across independent samples. However, the mito-ribo ratio was significantly elevated in droplets identified as doublets and/or multiplets (Figure 1G, p < 1e−10), supporting this as an informative metric to identify doublets and/or multiplets in combination with other droplet features.

**Figure 2:**
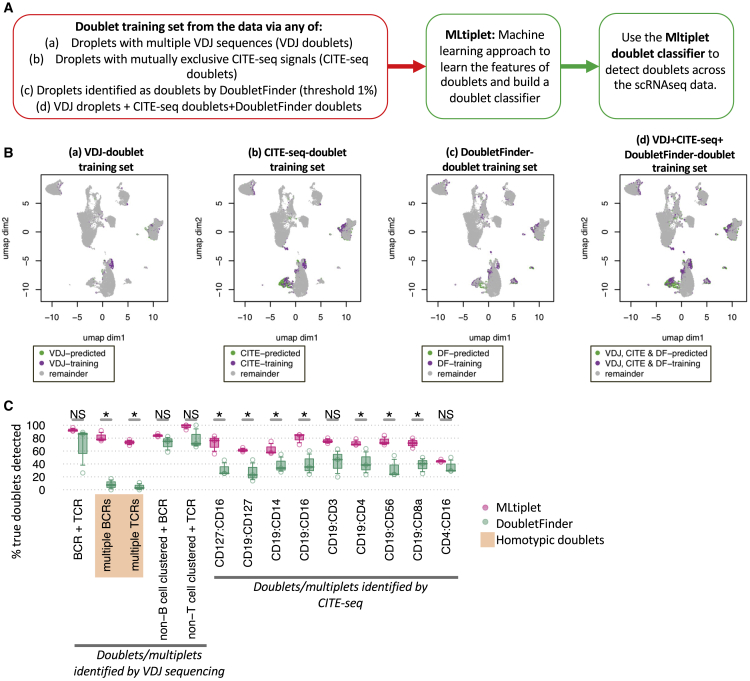
Machine learning applied to doublets and/or multiplet training data captures both homotypic and heterotypic doublets and/or multiplet (A) Schematic of the MLtuplet applied to the healthy PBMC data. (B) UMAP dimensionality reductions of three healthy PBMCs colored by MLtiplet-predicted singlets and training and predicted doublets across the VDJ-seq, CITE-seq, DoubletFinder training sets, and a training set combining all three approaches. (C) The proportion of VDJ and CITE-seq identified (true) doublets and/or multiplets that were identified as doublets by MLtiplet and DoubletFinder, grouped by doublet type. The doublets highlighted in orange are homotypic doublets (comprised of multiple cells of similar transcriptional types, namely B or T cells). ∗p < 0.05 (two-way Wilcox test, corrected for multiple testing). p values are calculated between the MLtiplet-predicted doublets and/or multiplets and singlets by using two-sided Wilcoxon test, and ∗p < 0.05.

We then applied a logistic regression by using the generalized linear model to fit the profile of these *identified doublets and/or multiplets* compared with the remainder of the droplets (enriched for *true singlets*), using the mito-ribo ratio, the per-sample centred log-ratio transformed nUMI counts and the module scores for each cell type or cluster as model inputs (Figure S1A). The module score is a per-cell score representing the relative likelihood of a cell being a member of a particular cell type/cluster (using the Seurat *AddModuleScore* function of the top 5 differentially expressed genes for each cell type/cluster). This provides the model with parameters associated with cell-type mixing as a result of hybrid transcriptomes present in droplets containing more than one cell. This model was then used as a classifier to detect the remaining doublets and/or multiplets for which the CITE-seq and/or VDJ-seq was not available/appropriate (Figure S1A), named MLtiplet. This model approach allows for the input of different training doublet/multiplet datasets, depending on the data available (Figures 2A and 2B). This provides a doublet/multiplet probability for each droplet based on their droplet features (Figures S2H, S3A, and S3B). MLtiplet was able to identify 791, 2,332, and 2,283 doublets and/or multiplets in the healthy PBMCs by using the VDJ-seq, CITE-seq, and DF training sets, respectively, primarily in the “B cell CD5^+^GZMB^−^ memory,” “B cell CD5^−^GZMB^+^CD27^−^,” and “unconventional monocyte” clusters (Table S1).

Assuming that the *identified doublets and/or multiplets* are enriched for true doublets and/or multiplets, then the sensitivity of doublet detection might be quantified through the proportion of correctly labeled *identified doublets and/or multiplets* (Figure 2A). The sensitivity of doublet/multiplet detection of MLtiplet was significantly higher than the established DoubletFinder method (using default parameters, Figure 2C), both for heterotypic doublets and/or multiplets (such as those identified from CITE-seq or TCR-BCR discordance) and homotypic doublets and/or multiplets (such as those with multiple BCRs or TCRs). Indeed, DoubletFinder was only able to identify <20% of homotypic doublets and/or multiplets with multiple BCRs or TCRs, compared with >70% using MLtiplet. The droplet features of the predicted doublets and/or multiplets using MLtiplet have elevated numbers of nUMIs and mito-ribo ratios (Figures S3B–S3D). Furthermore, the differentially expressed genes between droplets predicted to be doublets and/or multiplets compared with those predicted to be singlets per cluster revealed signals of mixed-cell populations (Figures S3E and S3F), such as elevated CD3E and CD3D in the doublets that clustered with the B cells, TRBC2 CD69 and MS4A1 in the doublets that clustered with the monocytes/neutrophils.

In general, the larger the training dataset the greater statistical power for pattern recognition for doublet detection (Vabalas et al., 2019). Therefore, we determined the effect of different types and sizes of doublet/multiplet training datasets on the doublet prediction. Decreasing the number of droplets per training dataset also reduced the proportion of doublets and/or multiplets classified. However, using only 139 CITE-seq identified doublets and/or multiplets (comprising only 20% of VDJ-seq identified doublets and/or multiplets, Table S2) as the training set resulted in 435 droplets classified as doublets and/or multiplets (36% of that from the full VDJ-seq training set). Only including VDJ- and CITE-seq-identified doublets and/or multiplets from a single sample in the training set resulted in the prediction of, on average, 41% of droplets classified as doublets and/or multiplets compared with using total corresponding training set across all samples. This suggests that datasets without the VDJ-seq and/or CITE-seq modalities datasets can be combined with datasets from which some doublets and/or multiplets can be identified to predict the majority of doublets and/or multiplets through machine learning of the droplet features.

**Figure 3:**
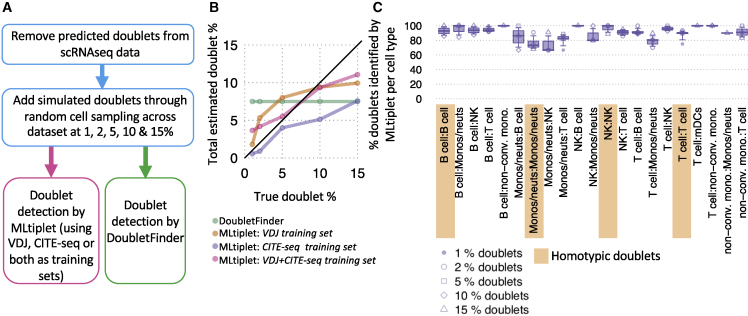
MLtiplet is both sensitive and specific on simulated doublets datasets, and scales with doublet proportion (A) Schematic of the comparison of the doublet detection methods using simulated data. (B) The estimated proportion of doublets across the simulated datasets using either DoubletFinder or the classifier based on VDJ-identified doublets, CITE-seq identified doublets, or both. The black line corresponds to y = x. (C) The percentages of doublets identified by MLtiplet per cell type across the different simulated datasets. The point shapes correspond to the simulated dataset for which the percentage of true doublets was either 1%, 2%, 5%, 10%, or 15%. The homotypic doublets are highlighted in orange.

Given that many currently used doublet detection methods require the prior estimation of multiplet rate, which is unknown in most scRNA-seq experiments, we compared the performance of MLtiplet and DoubletFinder on simulated data to assess how the doublet detection varies with true doublet proportions. Simulated RNA-seq, CITE-seq, and VDJ-seq data were generated to contain 1%, 2%, 5%, 10%, or 15% doublets, containing both heterotypic and homotypic doublets (see the STAR Methods, Figure 3A). Indeed, using the default parameters for DoubletFinder (with a prior of 7.5% doublets), the estimated doublet proportion remained constant irrespective of the true doublet proportion (Figure 3B). However, using either the VDJ-seq-identified doublets, CITE-seq-identified doublets, or both, as the training sets for MLtiplet, the estimated proportion of doublets scaled with the true proportion, with combined “VDJ-seq- and CITE-seq-identified doublets training set” performing best. Furthermore, MLtiplet accurately detected both heterotypic and homotypic doublets at a rate of >68% across all doublet combinations (Figure 3C). Therefore, we show that VDJ-seq- and CITE-seq-identified doublets might be used individually or in tandem for scalable detection of doublets and/or multiplets without prior knowledge of doublet proportions, and these methods outperform current doublet detection methods.

**Figure 4:**
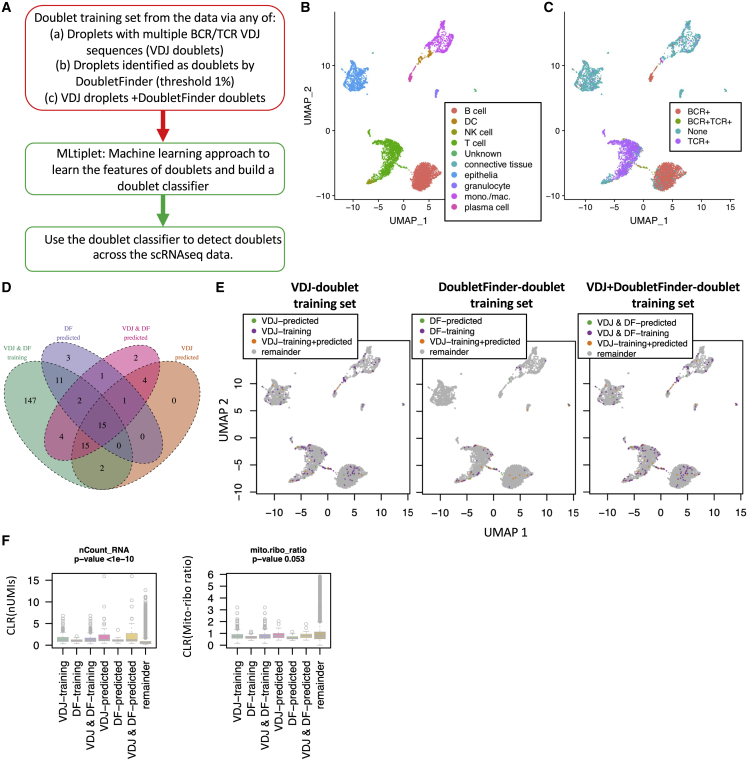
Validation of MLtiplet on an NSCLC tumor dataset Doublet detection on a non-small cell lung cancer (NSCLC) dataset. (A–C) Shown are the (A) schematic of the training datasets for doublet/multiplet prediction by MLtiplet. UMAP plots of (B) the annotated cell types and (C) VDJ-seq heterotypic doublets. (D) Venn diagram showing the numbers of droplets used as the combined identified doublets and/or multiples using both DoubletFinder and VDJ-seq (green), and the predicted doublets and/or multiplets from MLtiplet using the DoubletFinder-derived training dataset (blue), VDJ-seq-derived training dataset (orange), and DoubletFinder plus VDJ-seq-derived training dataset (pink). (E) UMAP plots of the training and predicted doublets and/or multiples using each approach. (F) The relative numbers of RNA molecules (nUMI) and mito-ribo ratio (mitoribo_ratio) per cell for the VDJ-identified doublets and/or multiplets, CITE-seq-identified doublets and/or multiplets, MLtiplet-predicted doublets and/or multiplets, and the remainder (predicted singlets by MLtiplet).

We next tested this approach on a sample derived from solid tissue, namely from a non-small cell lung cancer dataset (https://support.10xgenomics.com/single-cell-vdj/datasets, Figure 4). Here, we compare different input training doublet datasets for MLtiplet using VDJ-seq-identified doublets and/or multiplets, DoubletFinder-identified doublets and/or multiplets, or a combination of the two (Figure 3A). The three approaches identified similar distributions of doublets and/or multiples, with the combined input training datasets resulting in the highest number of predicted doublets (Figure 3B). Although we show that the number of doublets and/or multiplets identified in the B and T cell clusters are lower using DoubletFinder than using VDJ-seq (Table S3), running these doublet and/or multiplet training datasets through MLtiplet resulted in comparable numbers of predicted doublets and/or multiplets in each cell-type cluster. Differential gene expression analysis between the predicted doublets and/or multiplets compared with the predicted singlets per droplet cluster (Figure S3G) confirmed the mixed transcriptomic profiles of the predicted doublets and/or multiplets. This is exemplified by the elevated CD3D and CD3E expression in predicted doublets and/or multiplets within the "B cell", “DC,” and “plasma cell” clusters, which is indicative of T cell contamination. This demonstrates that the input training datasets for MLtiplet might be broadly generalizable to other inputs when VDJ-seq or CITE-seq are not be available. We further demonstrate this approach on a murine dataset derived from the PBMCs from two mouse strains (BALB/c and C57BL/6, https://support.10xgenomics.com/single-cell-vdj/datasets) (Figures S4A–S4F).

We also observed an enrichment of doublets and/or multiplets in specific cell clusters: (1) the majority of the “non-conventional monocyte” cluster were identified as doublets and/or multiplets in the healthy PBMC dataset (Figure 1D), suggesting that this cluster predominantly represents a subset of related hybrid transcriptomes. (2) Both homotypic doublets (containing >1 BCR chain type) and heterotypic doublets (containing BCRs and TCRs) were observed in the B cell cluster, suggesting that B-T cell hybrid transcriptome co-cluster with the true singlet B cell transcriptome populations. It is noted that there is an enrichment of some IGHV genes within the doublet droplets, suggesting that these cell-cell doublets are potentially as a result of cell-cell interactions that are enriched for certain antigen specificities (Figure S4G).

## Discussion

Overall, we demonstrate a doublet/multiplex droplet detection approach by using machine learning to predict doublets based on the features associated with identified hybrid droplets that can be applied to scRNA-seq datasets containing only VDJ-seq or CITE-seq information, or both. In theory, this might also be applied to scRNA-seq-only datasets through the identification of gene expression profiles that resemble hybrid transcriptomes, such as those identified through Scrublet or DoubletFinder to then feed into the doublet prediction model. The deficiency of previous doublet prediction methods for identifying homotypic doublets and/or multiplets is highlighted here by the CITE-seq and VDJ-seq analyses, and it is recommended to leverage these multi-omic single-cell data types for the generation of high-quality datasets. Thus, this presents a powerful approach, particularly for inflammatory cell-dominant scRNA-seq samples, to ensure high-quality scRNA-seq minimizing false discoveries of rare cell types from both homotypic and heterotypic doublets and/or multiplets or differential gene expression signatures, and finally ensuring the reproducibility of biological findings.

### Limitations of the study

Although identification of unseen homotypic and heterotypic cell multiplets using MLtiplet appears both sensitive and specific in our hands, the *a priori* assumptions do pose some limitations. Firstly, the definitive validation of mutually exclusive markers in the literature remains in evolution. Modeling multimodal cell doublets and/or multiplets, first required the curation of mutually exclusive CITE-seq markers and VDJ-seq chains based on best evidence to date (CD19 + CD3, CD19 + CD4, CD19 + CD8a, CD19 + CD56, CD19 + CD16, CD19 + CD14, CD19 + CD127, CD19 + CD56, CD4 + CD16, CD127 + CD16, STAR Methods). However, as the granularity of single-cell characterization improves, novel bona fide mixed-phenotypes arising from cellular plasticity or invariant VDJ-seq allelic inclusion might be revealed. To counter this limitation, we designed MLtiplet to allow the user to make heuristic decisions regarding which mutually exclusive markers to feed into the model, thus futureproofing for novel discoveries. Secondly, biological differences that might affect the absolute number of transcripts per cell, such as the physical cell size and proliferation status, cannot be modeled reliably. The use of nUMIs counts assumes a degree of homogeneity across these variables at the true single-cell level; therefore, physically large cells that are comparable in size to true multiplets might be incorrectly classified as doublets. Indeed, this is also a limitation for traditional exclusion of potential multiplets or poor-quality cells by thresholding on nUMIs, therefore it remains an unaddressed issue of cell size identification at the pre-bioinformatic level. Thirdly, due to the predominant use or commercial availability of CITE-seq antibodies targeting inflammatory cell markers, we have only been able to benchmark MLtiplet for identification of cell multiplets within these limitations. However, in theory, MLtiplet might take any custom barcode-conjugated antibodies targeting cell-surface markers that are mutually exclusive between two or more cell types. Finally, our proposed method's performance scales proportionally to the breadth of multi-omic data available. Although single-cell experiments are becoming more cost-effective, they remain prohibitively expensive with the addition of other modalities. Where mutually exclusive markers and/or VDJ chains are not present in the dataset, the sensitivity and specificity of the model reduces and thus this is a limitation that is proportional to the available resources at hand.

## STAR★Methods

### Key resources table

**Table:** 

REAGENT or RESOURCE	SOURCE	IDENTIFIER
**Deposited data**		

10X 5′ GEX + VDJ-seq of healthy human PBMCs	https://support.10xgenomics.com/single-cell-vdj/datasets	vdj_v1_hs_pbmc
10X 5′ GEX + VDJ-seq of healthy human PBMCs	https://support.10xgenomics.com/single-cell-vdj/datasets	vdj_v1_hs_pbmc2
10X 5′ GEX + VDJ-seq of healthy human PBMCs	https://support.10xgenomics.com/single-cell-vdj/datasets	vdj_v1_hs_pbmc3
10X 5′ GEX + VDJ-seq of human NSCLC sample	https://support.10xgenomics.com/single-cell-vdj/datasets	vdj_v1_hs_nsclc
10X 5′ GEX + VDJ-seq of healthy mouse PBMCs	https://support.10xgenomics.com/single-cell-vdj/datasets	vdj_v1_mm_c57bl6_pbmc
10X 5′ GEX + VDJ-seq of healthy mouse PBMCs	https://support.10xgenomics.com/single-cell-vdj/datasets	vdj_v1_mm_balbc_pbmc

**Software and algorithms**		

DoubletFinder	https://www.sciencedirect.com/science/article/pii/S2405471219300730#sec4	https://github.com/chris-mcginnis-ucsf/DoubletFinder
Seurat	https://www.nature.com/articles/nbt.3192	https://github.com/satijalab/seurat
MLtiplet	This paper	https://github.com/rbr1/MLtiplet

### Resource availability

#### Lead contact

Further information and requests for resources and reagents should be directed to and will be fulfilled by the Lead Contact, Rachael Bashford-Rogers (rbr1@well.ox.ac.uk).

#### Materials availability

This study did not generate new unique reagents.

#### Data and code availability

The code generated during this study are available at https://github.com/Bashford-Rogers-lab/MLtiplet. Original scRNA-seq data for the paper is available from https://support.10xgenomics.com/single-cell-vdj/datasets.

### Method details

#### scRNA-seq pre-processing and batch correction

The 10X Genomics’ Chromium scRNA-seq output data from three healthy donors’ PBMCs (https://support.10xgenomics.com/single-cell-vdj/datasets) was merged using the *Seurat* package in R. First, we filtered low-quality cells using Seurat (version 2.3.4), retaining cells with detected gene numbers >500, detected number of RNA molecules (>1000 unique molecular identifiers (UMIs) and <25% mitochondrial UMIs). We then normalised the gene counts for each cell using the *ScaleData* function in *Seurat* with the default parameters. Integration and batch correction were performed using the *harmony* package(Korsunsky et al., 2018) with default parameters, and using sample ID as a variable to regress out. The resulting harmony embeddings were used in the data visualized using a Uniform Manifold Approximation and Projection (UMAP) projection and subsequent clustering of cell types.

#### CITE-seq-identified doublet/multiplet training set

The raw CITE-seq data were normalised through centred log-ratio transformed (CLR) per sample (Figure S1B). Cells/droplets that were positive for each CITE-seq antibody were determined using the following method (Figures S1B and S2): for each CITE-seq antibody, the normalised CITE-seq levels between cell populations with high corresponding gene expression (such as T cells for CD3) and low corresponding gene expression (such as B cells and myeloid cells for CD3) were the input into a linear classifier (linear discriminant analysis, LDA). This was then used to determine the optimal threshold for distinguishing the threshold between CITE-seq positive and CITE-seq negative cells/droplets. Each cell/droplet was then classified to determine whether they are positive or negative. This was performed for each CITE-seq antibody. Mutually exclusive CITE-seq markers for the healthy PBMC dataset was: CD19 + CD3, CD19 + CD4, CD19 + CD8a, CD19 + CD56, CD19 + CD16, CD19 + CD14, CD19 + CD127, CD19 + CD56, CD4 + CD16, CD127 + CD16. Downstream analyses were performed on only the CITE-seq probes with classification of corresponding gene expression positive cells with a sensitivity of >70%.

The training set using the CITE-seq identification therefore relies on the co-positivity of mutually exclusive cell marker antibodies (cell-type-specific mutually exclusive CITE-seq pairs). This mutually exclusive list can be determined through either a literature search or comparison of the gene expression/CITE-seq levels between cell subtypes.

#### VDJ-seq-identified doublet/multiplet training set

The raw VDJ sequencing was filtered to remove VDJ sequences with non-productive sequences and fewer than 6 UMIs. VDJ-seq-identified doublets/multiplets were defined as droplets containing either (a) a BCR chain (IGH and/or IGK/L) and a TCR chain (TRA and/or TRB), (b) multiple BCRs (2 or more IGH chains and/or 2 or more IGK/L chains), (c) multiple TCRs (2 or more TRA chains and/or 2 or more TRB chains), (d) droplets that do not co-cluster with B cells via gene expression and contain a BCR chain (IGH and/or IGK/L), and (e) droplets that do not co-cluster with T cells via gene expression and contain a TCR chain (TRA and/or TRB).

#### Mito-ribo ratio

As a discriminant of singlets from doublets/multiplets, the mito-ribo ratio was used as a covariate, denoted by:

$$mito.ribo\;ratio=\frac{\sum m}{\sum \left(m+r\right)}$$

Where *m* is the per cell mtRNA UMI percentage and *r* is the rRNA UMI percentage.

#### MLtiplet model fitting

A generalised linear model was used to fit the profile of these *identified doublets/multiplets* compared to the remainder of the droplets (enriched for *true singlets*), using the mito-ribo ratio, the per-sample CLR transformed nUMI counts and the module scores for each cell type as model inputs. The module score is a per-cell score representing the relative likelihood of a cell being a member of a particular cell type (using the Seurat *AddModuleScore* function of the top 5 differentially expressed genes for each cell type). This provides the model with parameters associated with cell-type mixing as a result of hybrid transcriptomes present in droplets containing more than one cell. First the *identified doublets/multiplets* and the remainder of the cells were each filtered to include only those within 2 standard deviations of the mean for each variable used (mito-ribo ratio and per-sample CLR transformed nGenes, nUMIs, and VDJ UMI counts). Then the generalised linear model was fitted in R using *glm* function using logistic regression, and used to predict doublets across the total dataset.

#### Simulated data containing known proportions of doublets

The doublet simulated data were based on the scRNA-seq, CITE-seq and VDJ dataset from the 3 healthy PBMC samples. First, the experimental data were “cleaned” to remove droplets with nUMI counts > median + interquartile range per sample. Then simulated doublets were introduced through random cell sampling and sum their gene expression, VDJ BCR UMI, TCR UMI and CITE-seq counts, and were classified with the combined original cell type annotation labels. 5 simulated datasets were generated that incorporated either 1, 2, 5, 10 or 15% simulated doublets. Each simulated dataset was then pre-processed as described in “scRNA-seq pre-processing and batch correction”, and doublet detection methods were applied to the resulting datasets as described. MLtiplet accuracy of detected both heterotypic and homotypic doublets was calculated on all outputs for which there were >5 doublets.

### Quantification and statistical analyses

Statistical analyses were performed with R (https://www.r-project.org/). Data were presented as mean ± interquartile range. A p value of less than 0.05 was considered significant. ∗ p ≤ 0.05; ∗∗ p ≤ 0.005, ∗∗∗ p ≤ 0.0005. Statistical tests were performed via ANOVA for comparing differences in nUMIs, nGenes, or mito-ribo ratio between groups of cells. Heatmaps of gene expression and fold change between singlets and predicted doublets/multiplets were made with DoHeatmap in Seurat in R. Volcano plots of log p value against log fold change were done using ggplot2.

## Supplemental information

Document S1. Figures S1–S4 and Tables S1–S3

Document S2. Article plus supplemental information
