# Supplementary material for: Double-jeopardy: scRNA-seq doublet/multiplet detection using multi-omic profiling
Source: Cell Rep Methods. 2021 May 12;1(1):100008. doi: 10.1016/j.crmeth.2021.100008 (PMC8262260; doi:10.1016/j.crmeth.2021.100008)
Supplement: Document S1. Figures S1–S4 and Tables S1–S3 [file mmc1.pdf]

Cell Reports Methods, Volume *1*

Supplemental information

Double-jeopardy: scRNA-seq doublet/multiplet detection using multi-omic profiling

Bo Sun, Emmanuel Bugarin-Estrada, Lauren Elizabeth Overend, Catherine Elizabeth Walker, Felicia Anna Tucci, and Rachael Jennifer Mary Bashford-Rogers

Supplementary tables

Table S1. The numbers of doublets/multiplets doublets by VDJ-seq and CITE-seq per sample. Related to Figure 2.

		By sample			By gene expression type						
	**Doublet type**	**HC 1**	**HC 2**	**HC 3**	**B cells**	**Monocytes/ neutrophils**	**NK**	**T cells**	**mDCs**	**non-conv. monocytes**	**pDCs**
**Identified by VDJ-seq**	IGH+IGK/L+TRA or TRB	0	1	2	3	0	0	0	0	0	0
	3 of [IGH,IGK/L,TRA,TRB]	18	19	43	79	1	0	0	0	0	0
	IGH or IGK/L + TRA or TRB	22	23	54	95	1	0	3	0	0	0
	2x IGHs	54	12	29	93	2	0	0	0	0	0
	2x IGK/L	111	43	59	205	6	0	1	0	0	1
	2x TRBs	9	16	34	0	0	1	57	0	1	0
	2x IGH and 2x IGK/L	27	9	20	56	0	0	0	0	0	0
	2x TRAs and 2x TRBs	0	0	0	0	0	0	0	0	0	0
	non-B cell clustered + BCR	66	34	57	0	114	3	20	4	12	4
	non-T cell clustered + TCR	48	120	221	100	112	138	0	11	27	1
	**Total identified by VDJ-seq**	**253**	**210**	**372**	**333**	**225**	**141**	**77**	**15**	**39**	**5**
**Identified by CITE-seq**	CD127:CD16	95	118	283	16	110	262	26	1	81	0
	CD19:CD127	274	203	274	595	103	4	42	0	6	1
	CD19:CD14	92	40	71	16	162	3	13	1	6	2
	CD19:CD16	46	34	53	48	53	6	11	0	15	0
	CD19:CD3	124	91	170	285	51	3	44	0	1	1
	CD19:CD4	185	90	179	232	166	3	33	3	12	5
	CD19:CD56	38	46	51	64	46	6	17	0	2	0
	CD19:CD8a	62	40	72	91	46	4	31	0	2	0
	CD4:CD16	348	278	483	10	257	192	32	8	607	3
	**Total identified by CITE-seq**	**684**	**517**	**867**	**690**	**382**	**289**	**76**	**11**	**612**	**8**
**Identified by MLtiplet**	**VDJ-seq training set**	227	190	374	248	256	142	78	20	42	5
	**CITE-seq training set**	775	562	995	821	431	300	87	16	669	8
	**DF training set**	782	377	1124	393	585	173	222	358	398	154
	**Total droplets**	**7674**	**7024**	**11382**	**2982**	**5346**	**2461**	**13964**	**420**	**749**	**158**

Table S2. The number of predicted doublets using different doublet training sets for the healthy PBMC dataset. Related to Figure 2.

Training set	Number of droplets used in training set	Total doublets predicted	Total doublets predicted per sample			Total doublets predicted per cluster						
			**HC 1**	**HC2**	**HC3**	**B cells**	**Mono / neuts**	**NK**	**T cells**	**mDC s**	**non-conv. Mono .**	**pDCs**
**VDJ-training.0.2x**	139	435	142	105	188	289	82	10	5	3	40	6
**VDJ-training.0.4x**	278	640	209	165	266	423	123	16	18	28	29	3
**VDJ-training.0.6x**	416	843	263	218	362	535	167	48	36	22	27	8
**VDJ-training.0.8x**	555	1037	321	261	455	601	194	77	60	26	62	17
**VDJ-training.1x**	693	1219	371	309	539	736	221	98	73	27	53	11
**CITE-training.0.2x**	414	1891	595	455	841	1090	127	33	9	3	628	1
**CITE-training.0.4x**	828	2912	918	710	1284	1634	192	407	14	12	650	3
**CITE-training.0.6x**	1241	3671	1198	907	1566	2035	264	647	26	17	678	4
**CITE-training.0.8x**	1655	4079	1351	1035	1693	2385	328	634	32	17	679	4
**CITE-training.1x**	2068	4817	1561	1245	2011	2541	428	1083	56	21	684	4
**DoubletFinder-training.0.2x**	408	1374	453	318	603	260	175	6	3	257	521	152
**DoubletFinder-training.0.4x**	816	2021	723	456	842	551	361	87	10	308	553	151
**DoubletFinder-training.0.6x**	1224	2751	963	635	1153	889	518	261	24	331	575	153
**DoubletFinder-training.0.8x**	1632	3596	1245	853	1498	1143	678	626	41	342	611	155
**DoubletFinder-training.1x**	2040	4499	1521	1088	1890	1515	904	878	66	356	625	155
**VDJ & CITE-training.0.2x**	463	1772	551	415	806	943	150	10	5	18	642	4
**VDJ & CITE-training.0.4x**	926	2348	728	578	1042	1353	234	69	19	23	646	4
**VDJ & CITE-training.0.6x**	1389	3293	1069	812	1412	1885	356	289	29	61	663	10
**VDJ & CITE-training.0.8x**	1852	3947	1324	987	1636	2250	429	495	49	50	667	7
**VDJ & CITE-training.1x**	2314	4705	1559	1181	1965	2475	569	816	90	73	674	8
**VDJ, CITE & DoubletFinder-training.0.2x**	702	2222	755	512	955	784	288	134	11	240	613	152
**VDJ, CITE & DoubletFinder-training.0.4x**	1403	3844	1335	911	1598	1588	502	623	33	291	653	154
**VDJ, CITE & DoubletFinder-training.0.6x**	2104	5462	1799	1384	2279	2154	769	1313	76	326	670	154
**VDJ, CITE & DoubletFinder-training.0.8x**	2805	6589	2201	1689	2699	2603	1118	1554	131	343	686	154
**VDJ, CITE & DoubletFinder-training.1x**	3506	7453	2531	1896	3026	2788	1496	1772	187	365	691	154
**VDJ-training.HC_103**	139	570	325	96	149	433	114	2	7	2	11	1
**VDJ-training.HC_104**	278	439	120	125	194	219	83	41	21	15	56	4
**VDJ-training.HC_105**	416	596	113	177	306	302	113	76	30	21	32	22
**CITE-training.HC_103**	414	2283	877	511	895	1463	197	8	6	2	606	1
**CITE-training.HC_104**	828	2080	642	517	921	1127	162	99	16	13	658	5
**CITE-training.HC_105**	1241	3390	873	840	1677	1532	148	1002	39	9	655	5
**DoubletFinder-training.HC_103**	408	895	565	107	223	226	200	63	18	241	60	87
**DoubletFinder-training.HC_104**	816	1056	301	246	509	82	183	4	20	305	314	148
**DoubletFinder-training.HC_105**	1224	1667	353	452	862	456	365	32	25	297	341	151
**VDJ & CITE-training.HC_103**	463	2334	900	518	916	1479	227	6	5	2	614	1
**VDJ & CITE-training.HC_104**	926	1970	604	493	873	1018	176	71	18	29	654	4
**VDJ & CITE-training.HC_105**	1389	2746	758	661	1327	1243	214	570	37	25	649	8
**VDJ, CITE & DoubletFinder-training.HC_103**	702	2445	902	564	979	1102	401	14	18	253	541	116
**VDJ, CITE & DoubletFinder-training.HC_104**	1403	2213	723	534	956	837	279	38	31	242	643	143
**VDJ, CITE & DoubletFinder-training.HC_105**	2104	2930	880	703	1347	1065	475	260	54	300	635	141

Abbreviations:

VDJ-seq.*a*x (where *a = 0.2, 0.4, 0.6, 0.8, 1.0*) refers to VDJ-identified doublets in the training set with the proportion *a* randomly subsampled.

CITE-seq.*a*x refers to CITE-identified doublets in the training set with the proportion *a* randomly subsampled.
VDJ&CITE-seq.ax refers to VDJ and CITE-identified doublets in the training set with the proportion *a* randomly subsampled.
VDJ, CITE & DoubletFinder-training.ax refers to VDJ, CITE-seq and DoubletFinder-identified doublets in the training set with the proportion *a* randomly subsampled.
METHOD*.Sample*X (where *X = 1, 2, 3 and METHOD* = VDJ-seq., CITE-seq, VDJ&CITE-seq, VDJ, CITE & DoubletFinder-training) refers to the identified doublets in via the corresponding method for the the training set from only healthy PBMC sample *X*.

Table S3. The number of total droplets, and identified doublets/multiplets with different methods, and predicted doublets using different doublet training sets for the NSCLC dataset. Related to Figure 4.

		B cell	connective tissue	DC	epithelia	Granulo-cyte	mono./ mac.	NK cell	plasma cell	T cell	Unknown
	**All droplets**	**2327**	**20**	**154**	**1149**	**148**	**782**	**140**	**147**	**1813**	**48**
**MLtiplet training doublets/ multiplets**	VDJ training	26	0	23	19	2	18	4	8	65	3
	DF training	9	0	0	0	1	4	7	0	43	3
	VDJ DF training	33	0	23	19	3	22	7	8	75	6
**MLtiplet predictions**	VDJ predicted	3	0	4	4	0	2	2	4	16	2
	DF predicted	7	0	1	0	1	0	3	0	19	2
	VDJ DF predicted	3	0	5	4	1	2	2	5	20	2

Supplementary figures

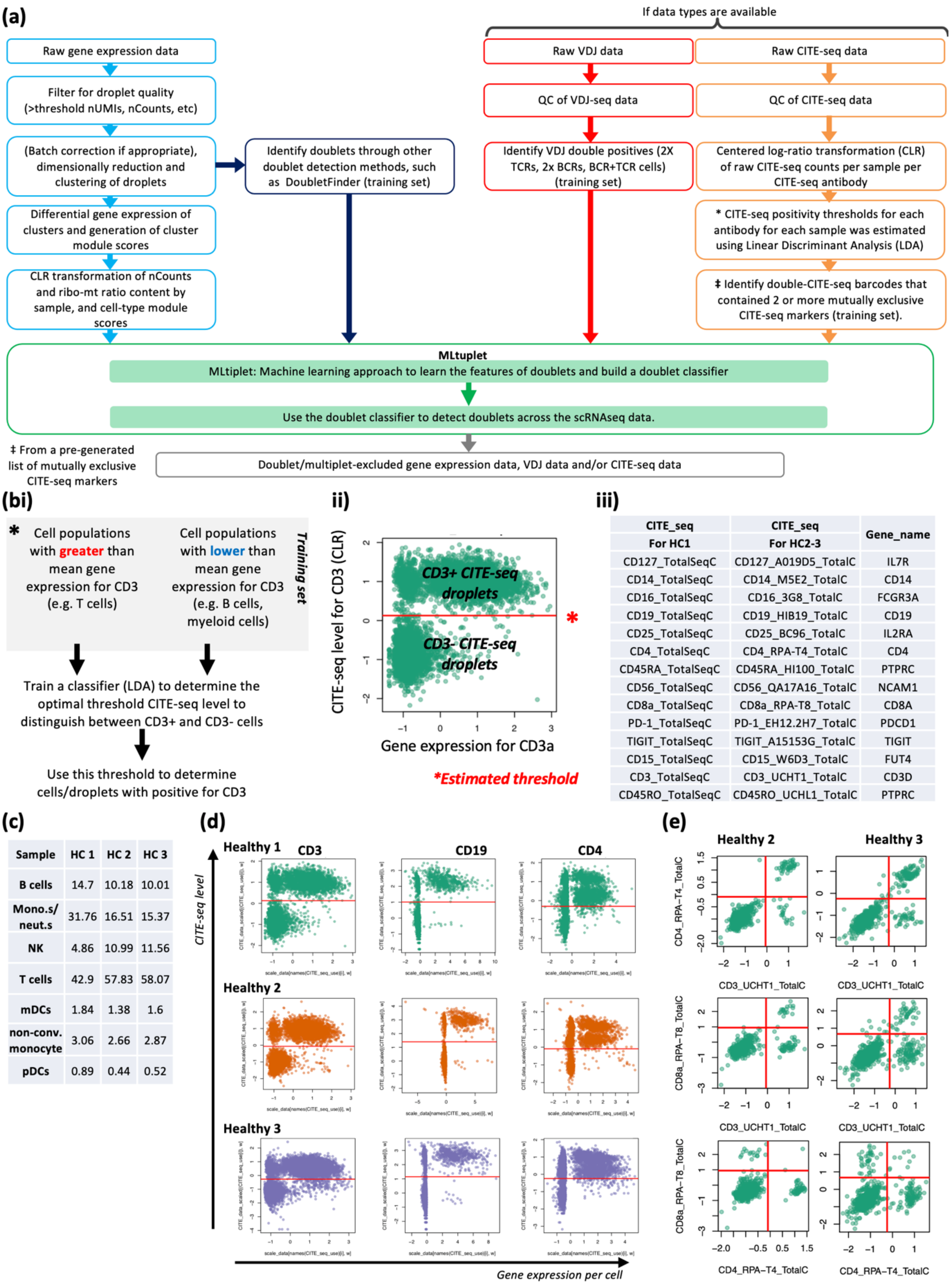

(biii)

CITE_seq For HC1	CITE_seq For HC2-3	Gene_name
CD127_TotalSeqC	CD127_A019D5_TotalC	IL7R
CD14_TotalSeqC	CD14_M5E2_TotalC	CD14
CD16_TotalSeqC	CD16_3G8_TotalC	FCGR3A
CD19_TotalSeqC	CD19_HIB19_TotalC	CD19
CD25_TotalSeqC	CD25_BC96_TotalC	IL2RA
CD4_TotalSeqC	CD4_RPA-T4_TotalC	CD4
CD45RA_TotalSeqC	CD45RA_HI100_TotalC	PTPRC
CD56_TotalSeqC	CD56_QA17A16_TotalC	NCAM1
CD8a_TotalSeqC	CD8a_RPA-T8_TotalC	CD8A
PD-1_TotalSeqC	PD-1_EH12.2H7_TotalC	PDCD1
TIGIT_TotalSeqC	TIGIT_A15153G_TotalC	TIGIT
CD15_TotalSeqC	CD15_W6D3_TotalC	FUT4
CD3_TotalSeqC	CD3_UCHT1_TotalC	CD3D
CD45RO_TotalSeqC	CD45RO_UCHL1_TotalC	PTPRC

(c)

Sample	HC 1	HC 2	HC 3
B cells	14.7	10.18	10.01
Mono.s/ neut.s	31.76	16.51	15.37
NK	4.86	10.99	11.56
T cells	42.9	57.83	58.07
mDCs	1.84	1.38	1.6
non-conv. monocyte	3.06	2.66	2.87
pDCs	0.89	0.44	0.52

Figure S1. Workflow of MLtiplet doublet/multiplet detection using VDJ-seq and CITE-seq modalities. Related to Figure 1.

(a) Schematic of MLtiplet doublet detection workflow. (bi) Detailed explanation of step CITE-seq positivity thresholds for each antibody for each sample (marked by a * in (a)) using CD3 as an example. For each CITE-seq antibody, the normalised CITE-seq levels between cell populations with high corresponding gene expression (such as T cells for CD3, bii) and low gene expression (such as B cells and myeloid cells) were the input into a linear classifier to determine the optimal threshold for distinguishing the CD3 CITE-seq positive and CD3 CITE-seq negative cells/droplets. Each cell/droplet is then classified to determine whether they are positive (CD3 CITE-seq level greater than threshold) or negative (CD3 CITE-seq level lower than threshold). This is performed for each CITE-seq antibody. (biii) The available CITE-seq probes from the peripheral blood mononuclear cells (PBMCs) from three healthy individuals (https://support.10xgenomics.com/single-cell-vdj/datasets). (c) The percentages of each broad immune cell type per sample annotated through differential gene expression and CITE-seq marker expression. (d) Examples of the CITE-seq thresholds set per patient for CITE-seq antibodies targeting CD3, CD19 and CD4. (e) Examples of the CITE-seq levels between CD3, CD4 and CD8 for the healthy individuals 2 and 3 for the B cell cluster, with the red lines corresponding to the CITE-seq positivity thresholds. The equivalent plot for healthy individual 1 is provided in Figure 1.

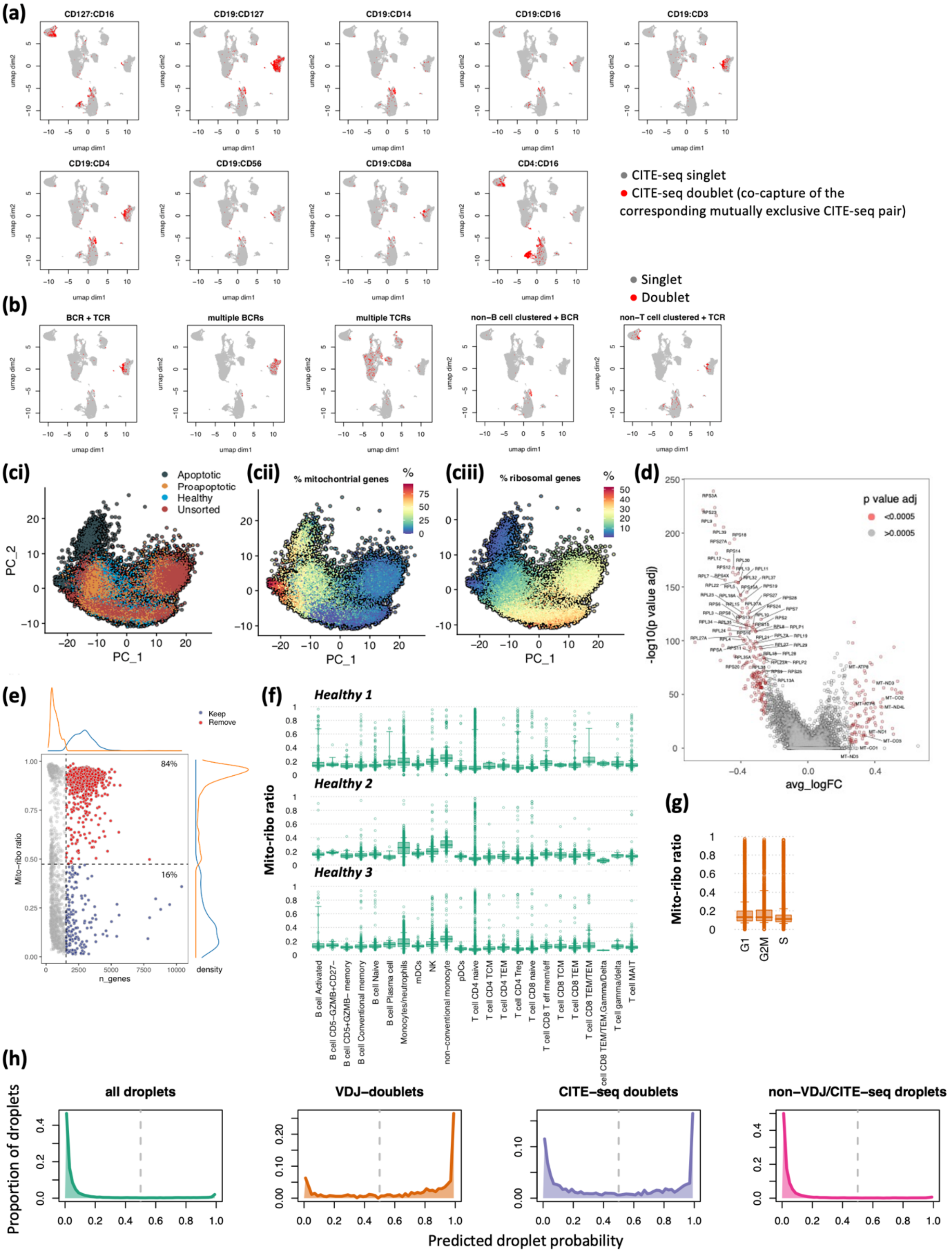
(a)
CD127:CD16
CD19:CD127
CD19:CD14
CD19:CD16
CD19:CD3
CD19:CD4
CD19:CD56
CD19:CD8a
CD4:CD16
CITE-seq singlet
CITE-seq doublet (co-capture of the corresponding mutually exclusive CITE-seq pair)
Singlet
Doublet
(b)
BCR + TCR
multiple BCRs
multiple TCRs
non−B cell clustered + BCR
non−T cell clustered + TCR
(ci)
Apoptotic
Proapoptotic
Healthy
Unsorted
PC_1
PC_2
(cii)
% mitochontrial genes
(ciii)
% ribosomal genes
(d)
p value adj
<0.0005
>0.0005
-log10(p value adj)
avg_logFC
(e)
Keep
Remove
84%
16%
Mito−ribo ratio
n_genes
density
(f)
Healthy 1
Healthy 2
Healthy 3
Mito-ribo ratio
B cell Activated
B cell CD5−GZMB+CD27−
B cell CD5+GZMB− memory
B cell Conventional memory
B cell Naive
B cell Plasma cell
Monocytes/neutrophils
mDCs
NK
non−conventional monocyte
pDCs
T cell CD4 naive
T cell CD4 TCM
T cell CD4 TEM
T cell CD4 Treg
T cell CD8 naive
T cell CD8 T eff mem/eff
T cell CD8 TCM
T cell CD8 TEM
T cell CD8 TEM/TEM
T cell CD8 TEM/TEM.Gamma/Delta
T cell gamma/delta
T cell MAIT
(g)
Mito-ribo ratio
G1
G2M
S
(h)
all droplets
VDJ−doublets
CITE−seq doublets
non−VDJ/CITE−seq droplets
Proportion of droplets
Predicted droplet probability

Figure S2. Doublet/multiplet detection using VDJ-seq and CITE-seq modalities in human healthy PBMCs and MLtiplet training features.
Related to Figure 1.

(a) The UMAP distributions of the CITE-seq doublet droplets (red) and remainder droplets (grey). (b) The UMAP distributions of the BCR/TCR doublet droplets (red) and remainder droplets (grey). (ci) PCA plot of HEK293 cells enriched for apoptotic, proapoptotic, healthy and unsorted populations. (cii) PCA coloured by percentage mitochondrial gene counts of total gene counts. (ciii) PCA coloured by percentage ribosomal gene counts of total gene counts. (d) Differential gene expression analysis between apoptotic and healthy single cells. (e) Mito-ribo ratio vs. total number of genes detected with corresponding density plots. Inferred doublets assigned to be removed are highlighted in red. Percentages shown are calculated on all cells above the local minimum of a GMM fitted on total genes detected, represented by the dotted vertical line. The mito-ribo ratios for each droplet across (f) cell types and (g) cell cycle phases per healthy PBMC sample (as defined by gene expression). (h) Histogram of doublet prediction probabilities by MLtiplet for (from left to right) all droplets, doublets/multiplets identified by VDJ-seq, doublets/multiplets identified by CITE-seq, and droplets that were not identified as doublets/multiplets identified by VDJ-seq or CITE-seq.

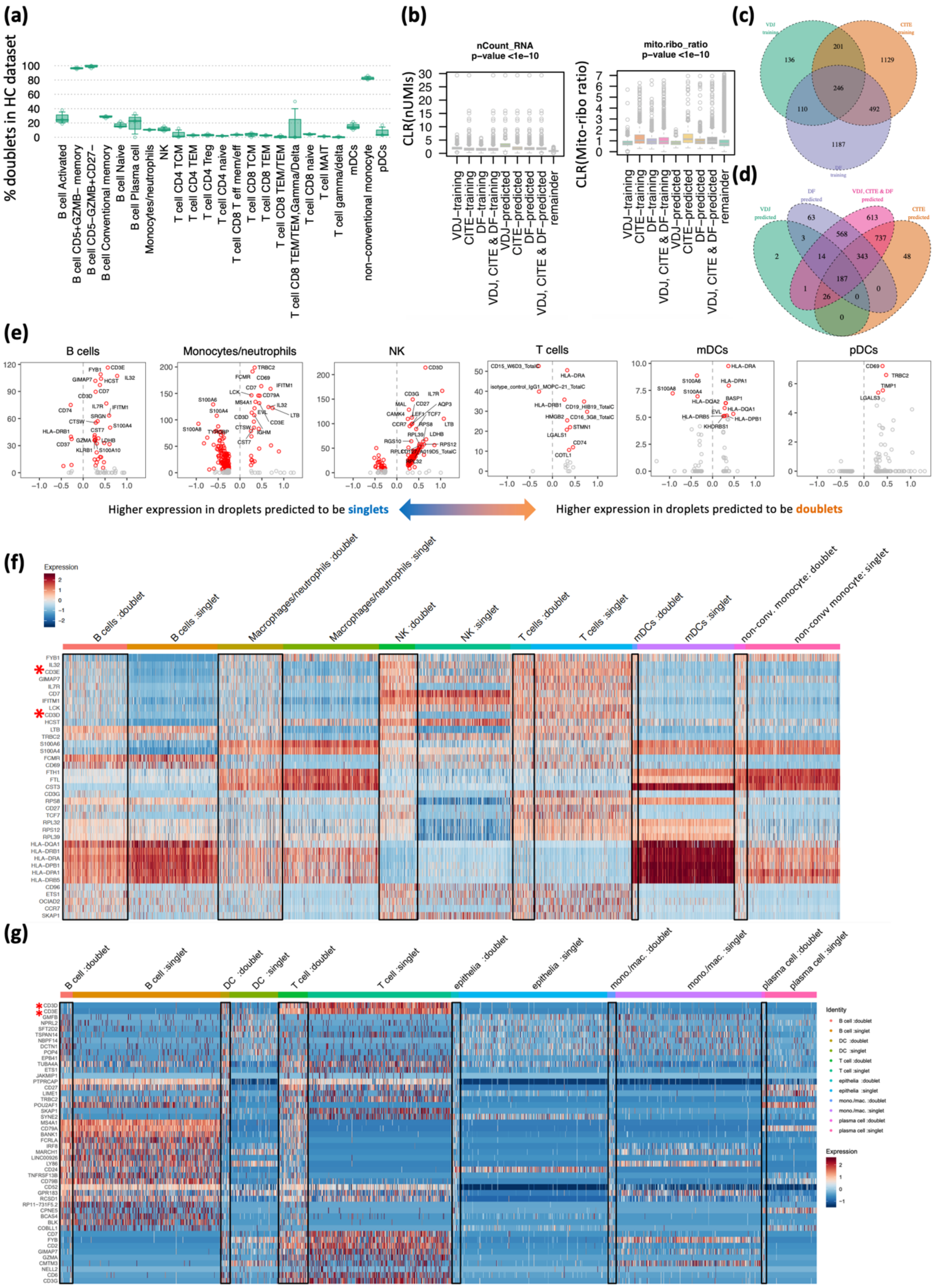
(a)
% doublets in HC dataset
B cell Activated
B cell CD5+GZMB− memory
B cell CD5−GZMB+CD27−
B cell Conventional memory
B cell Naive
B cell Plasma cell
Monocytes/neutrophils
NK
T cell CD4 TCM
T cell CD4 TEM
T cell CD4 Treg
T cell CD4 naive
T cell CD8 T eff mem/eff
T cell CD8 TCM
T cell CD8 TEM
T cell CD8 TEM/TEM
T cell CD8 TEM/TEM,Gamma/Delta
T cell CD8 naive
T cell MAIT
T cell gamma/delta
mDCs
non-conventional monocyte
pDCs
(b)
nCount_RNA
p-value <1e−10
CLR(nUMIs)
mito.ribo_ratio
p-value <1e−10
CLR(Mito-ribo ratio)
VDJ−training
CITE−training
DF−training
VDJ, CITE & DF−training
VDJ−predicted
CITE−predicted
DF−predicted
VDJ, CITE & DF−predicted
remainder
(c)
VDJ training
CITE training
DF training
(d)
VDJ predicted
DF predicted
VDJ, CITE & DF predicted
CITE predicted
(e)
B cells
Monocytes/neutrophils
NK
T cells
mDCs
pDCs
Higher expression in droplets predicted to be singlets
Higher expression in droplets predicted to be doublets
(f)
Expression
B cells :doublet
B cells :singlet
Macrophages/neutrophils :doublet
Macrophages/neutrophils :singlet
NK :doublet
NK :singlet
T cells :doublet
T cells :singlet
mDCs :doublet
mDCs :singlet
non-conv. monocyte: doublet
non-conv monocyte: singlet
(g)
B cell :doublet
B cell :singlet
DC :doublet
DC :singlet
T cell :doublet
T cell :singlet
epithelia :doublet
epithelia :singlet
mono./mac. :doublet
mono./mac. :singlet
plasma cell :doublet
plasma cell :singlet
Identity
Expression

Figure S3. Features of training and predicted doublet/multiplet sets.
Related to Figure 2.

(a) Percentages of predicted doublets by MLtiplet per cell annotation in healthy scRNA-seq dataset. (b) The relative numbers of RNA molecules (nUMI) and mito-ribo ratio (mitoribo_ratio) per droplet for the VDJ-identified doublets/multiplets, the CITE-seq-identified and DoubletFinder (DF)-identified doublets/multiplets (training) and the resulting predicted doublets/multiplets derived from these training datasets using MLtuplet. "Remainder" refers to droplets that were not identified/predicted to be doublets/multiplets from the VDJ-seq or CITE-seq data. The p-values of the differences between the feature distributions of the doublet/multiplets detected and the remainder of the droplets provided (two-sided Wilcoxon test). Venn diagrams of the numbers of droplets in (c) each training set by method, and (d) predicted from MLtuplet using each training set. (e) Volcano plots of the differential gene expression between droplets predicted to be doublets/multiplets compared to those predicted to be singlets per UMAP cluster. The top 20 genes with a p-value <1e5 are labelled. (f) Heatmap of differential gene expression between healthy PBMC between predicted singlets and doublets/multiplets per cell type cluster. (g) Heatmap of differential gene expression between predicted singlets and doublets/multiplets per cell type cluster.

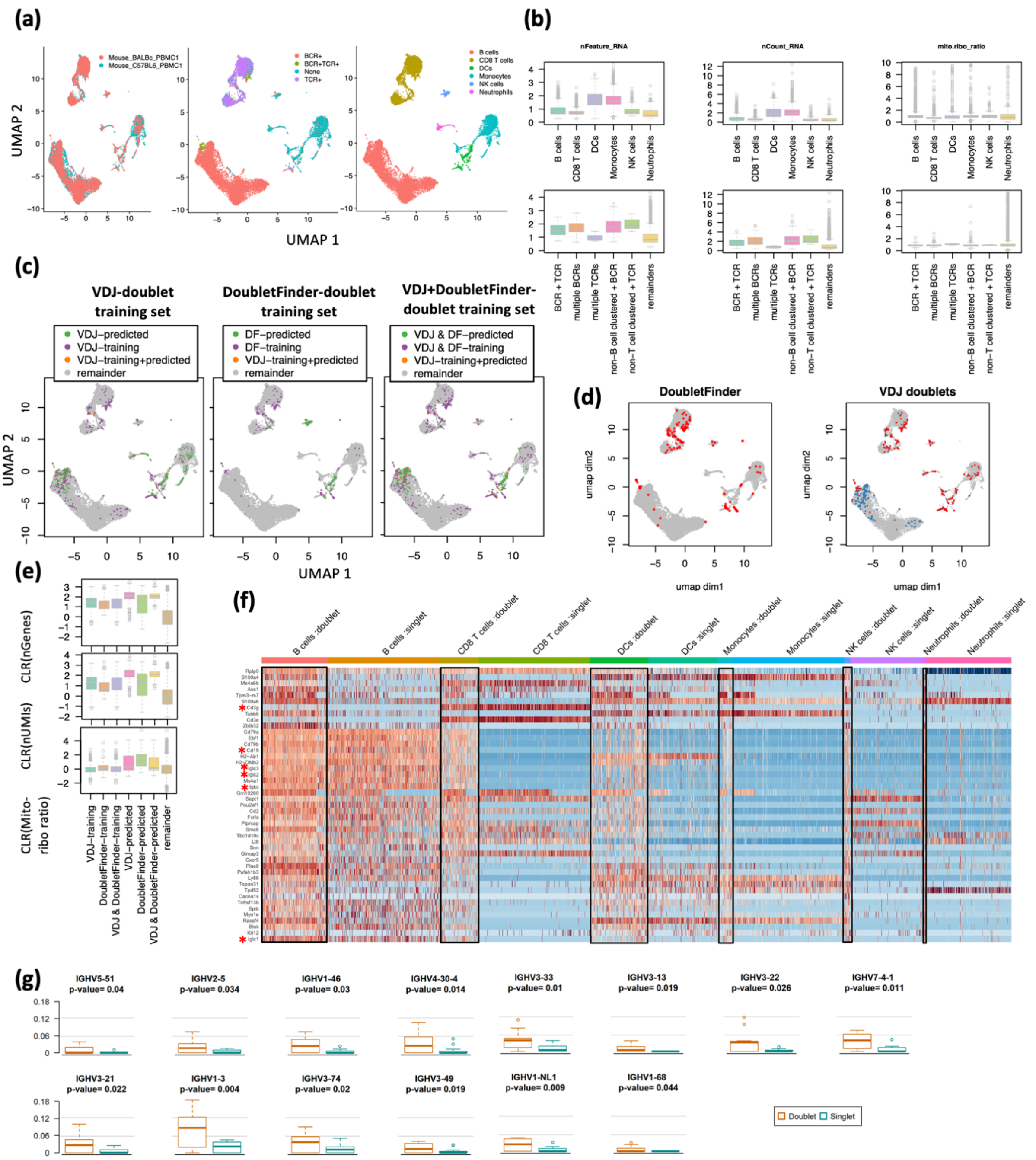

Figure S4. MLtiplet training features and predictions from a murine PBMC dataset. Related to Figure 4.

Doublet detection on a murine dataset comprising PBMCs from two mouse strains (BALB/c and C57BL/6. (a) UMAP plots of (left) each mouse sample, (middle) VDJ-seq information, and (right) the annotated cell types. (b) The relative numbers of genes (nFeatures), RNA molecules (nUMI) and mito-ribo ratio (mitoribo_ratio) per cell for (top) each cell type, and (bottom) the VDJ-identified doublets/multiplets. (c) UMAP plot of the doublets identified from (left) DoubletFinder and (right) VDJ-seq heterotypic doublets. (d) The relative numbers of genes (nFeatures), RNA molecules (nUMI) and mito-ribo ratio (mitoribo_ratio) per cell for the VDJ-identified doublets/multiplets, DoubletFinder-identified doublets/multiplets, MLtiplet predicted doublets/multiplets and the remainder (predicted singlets by MLtiplet). (e) UMAP plots of the training and predicted doublets/multiples using each approach. (f) Heatmap of differential gene expression between murine PBMC between predicted singlets and doublets/multiplets per cell type cluster. (g) Enrichment of IGHV gene usages between doublets and singlets in three healthy peripheral blood samples. Tests performed by MANOVA in R.
