# Supplementary material for: Double-jeopardy: scRNA-seq doublet/multiplet detection using multi-omic profiling
Source: Cell Rep Methods. 2021 May 12;1(1):100008. doi: 10.1016/j.crmeth.2021.100008 (PMC8262260; doi:10.1016/j.crmeth.2021.100008)
Supplement: Document S2. Article plus supplemental information [file mmc2.pdf]

Cell Reports
Methods

Report

Double-jeopardy: scRNA-seq doublet/multiplet detection using multi-omic profiling

Graphical abstract

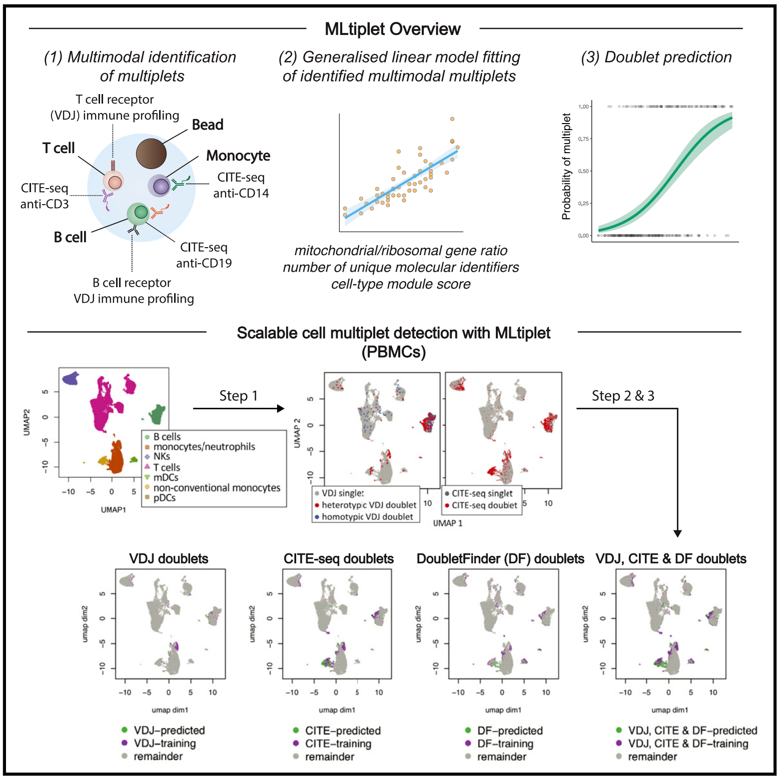

Highlights

- Doublets and multiplets in single-cell RNA-seq confound biological results
- Single-cell VDJ-seq and CITE-seq can aid the identification of doublets/multiplets
- Machine learning can identify further heterotypic and homotypic doublets/multiplets

Authors

Bo Sun, Emmanuel Bugarin-Estrada, Lauren Elizabeth Overend, Catherine Elizabeth Walker, Felicia Anna Tucci, Rachael Jennifer Mary Bashford-Rogers

Correspondence

bo.sun@ndcn.ox.ac.uk (B.S.),
rbr1@well.ox.ac.uk (R.J.M.B.-R.)

In brief

Sun et al. demonstrate that doublets/multiplets might be identified from single-cell RNA-seq datasets through analysis of mutually exclusive VDJ-seq and/or CITE-seq features. Moreover, we use machine learning approaches for doublet/multiplet detection utilizing VDJ-seq and/or CITE-seq information to predict their presence based on transcriptional features associated with identified hybrid droplets.

Sun et al., 2021, Cell Reports Methods *1*, 100008
May 24, 2021 © 2021 The Author(s).
https://doi.org/10.1016/j.crmeth.2021.100008

CellPress

Cell Reports Methods

CellPress
OPEN ACCESS

Report

Double-jeopardy: scRNA-seq doublet/multiplet detection using multi-omic profiling

Bo Sun,[1,2,*] Emmanuel Bugarin-Estrada,[1] Lauren Elizabeth Overend,[1] Catherine Elizabeth Walker,[1] Felicia Anna Tucci,[1] and Rachael Jennifer Mary Bashford-Rogers[1,3,*]

[1]Wellcome Centre for Human Genetics, University of Oxford, Oxford, UK

[2]Oxford Autoimmune Neurology Group, Nuffield Department of Clinical Neurosciences, University of Oxford, Oxford, UK

[3]Lead contact

*Correspondence: bo.sun@ndcn.ox.ac.uk (B.S.), rbr1@well.ox.ac.uk (R.J.M.B.-R.)

https://doi.org/10.1016/j.crmeth.2021.100008

MOTIVATION Single-cell RNA sequencing (scRNA-seq) techniques are transforming our understanding of multicellular organisms, disease states, and cellular heterogeneity. The aggregation of two or more cells into single droplets (doublets/multiplets) during the cell capture step of scRNA-seq resulting in hybrid transcriptomes can lead to false discoveries of rare cell types, intermediate cell states, and disease-associated transcriptomic signatures. Current methods do not sensitively identify many doublets/multiplets. Here, we address this doublet/multiplet detection issue utilizing VDJ-seq and/or CITE-seq, and apply machine learning to predict their presence based on transcriptional features associated with identified hybrid droplets, and ultimately scRNA-seq data quality.

SUMMARY

The computational detection and exclusion of cellular doublets and/or multiplets is a cornerstone for the identification the true biological signals from single-cell RNA sequencing (scRNA-seq) data. Current methods do not sensitively identify both heterotypic and homotypic doublets and/or multiplets. Here, we describe a machine learning approach for doublet/multiplet detection utilizing VDJ-seq and/or CITE-seq data to predict their presence based on transcriptional features associated with identified hybrid droplets. This approach highlights the utility of leveraging multi-omic single-cell information for the generation of high-quality datasets. Our method has high sensitivity and specificity in inflammatory-cell-dominant scRNA-seq samples, thus presenting a powerful approach to ensuring high-quality scRNA-seq data.

INTRODUCTION

The use of single-cell RNA sequencing (scRNA-seq) techniques has revolutionized the characterization of complex biological systems in health and diseased states; however, its fundamental success relies on the true representations of high-quality single cells. Droplet-based scRNA-seq technologies have allowed for the capture and analysis of 1,000s–100,000s of cells in single experiments at reduced per-cell cost. The success of single-cell experiments depends on the accurate capture of a single cell from a prepared cell suspension. However, many scRNA-seq techniques face significant limitations, including the capture of doublets (or multiplets) and/or low-quality or dying cells, which potentially confounds biological results. Doublets and multiplets are defined as the aggregation of two or more cells into single droplets during the cell capture step of scRNA-seq, resulting in hybrid transcriptomes (Zheng et al., 2017; Wolock et al., 2019; McGinnis et al., 2019). Hybrid transcriptomes might be derived from the aggregation of two or more intact cells and/or dying and/or broken cells and can be homotypic (comprised of cells that are transcriptionally similar) or heterotypic (comprised of cells with dissimilar gene expression). Conditions resulting in a propensity for cellular clumping might contribute to the formation of cellular doublets or the non-specific binding of detection antibodies. The aggregation of cells undergoing apoptosis is well described in flow cytometry experiments (Cui et al., 2016; Kuonen et al., 2010). Although most single-cell experiments perform dead cell exclusion, the protocols can be protracted and result in further cell death downstream. These processes can result in false discoveries of rare cell types, intermediate cell states and disease-associated transcriptomic signatures (Stegle et al., 2015; Ilicic et al., 2016). Although prospective experimental planning might reduce the frequency of doublet/multiplet occurrence, such as capturing fewer cells during scRNA-seq library

This is an open access article under the CC BY license (http://creativecommons.org/licenses/by/4.0/).

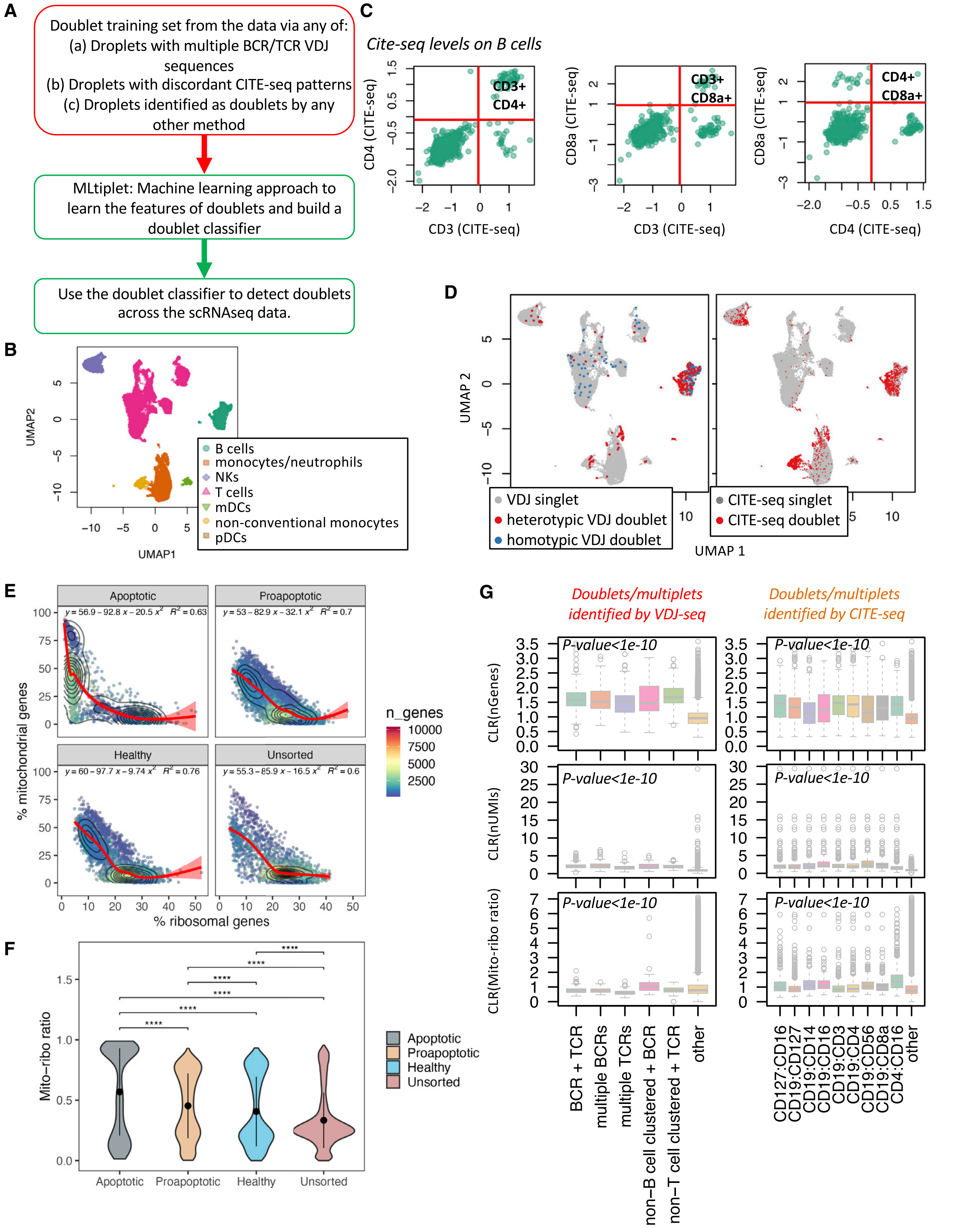

(legend on next page)

preparation, doublet and/or multiplet capture is a problem in all experimental setups (Zheng et al., 2017; Wolock et al., 2019; McGinnis et al., 2019).

The computational detection and exclusion of doublet or multiplet artifacts is required to accurately analyze the true biological signals in single-cell data. Doublet detection might be achieved experimentally through cell hashing (Stoeckius et al., 2018) (pooling of multiple samples labeled with distinct oligo-tagged antibodies against ubiquitously expressed surface proteins) or through genotype-based multiplexing (Kang et al., 2018) (genetic variation between multiplexed sample donors to determine the sample identity of each cell and detect droplets containing two or more cells); however, these data modalities are not suitable or included in many scRNA-seq experiments. Current methods for identification of same-sample doublets and/or multiplets include DoubletFinder (McGinnis et al., 2019), Scrublet (Wolock et al., 2019), DoubletDecon (Depasquale et al., 2019), and scds (Bais and Kostka, 2020), all of which primarily identify only heterotypic doublets, and some methods require the prior estimation of multiplet rate, which is not possible to evaluate (McGinnis et al., 2019). There are no robust identification methods for both heterotypic and homotypic doublets and/or multiplets.

Here, we describe approaches for the detection of both heterotypic and homotypic doublets and/or multiplets. This is possible through the use of recent multi-omic approaches in scRNA-seq experiments that characterize the proteomic (Stoeckius et al., 2017) and immune receptor profiles of single cells (Azizi et al., 2018) offering further modalities of multiplet identification that build upon current gene expression-based tools (Wolock et al., 2019; McGinnis et al., 2019). The use of barcoded oligonucleotide-conjugated antibodies that target cell-surface markers (CITE-seq) can potentially identify cell multiplets through the occurrence of co-staining for cell-type-specific canonical markers, such as CD3 and CD19, exclusive markers for T and B cells, respectively. Similarly, the expression of more than one clonally distinct T or B cell receptor (TCR or BCR, respectively) chain in a single T or B cell is a rare occurrence. In B cells, double light chains occur at 2%–10% in murine models (Casellas et al., 2007), whereas allelically inclusive expression of double heavy chains is incredibly rare at reported frequencies of 0.01% (Barreto and Cumano, 2000). In T cells, the expression of more than one distinct alpha chain (TCRα) has been estimated to occur in 1%–10% of human peripheral T cells (Padovan et al., 1993), whereas double beta chain (TCRβ) are rarer at frequencies of <1%. The possibility of a T or B cell endogenously expressing the respective cell-type-specific receptor (Ahmed et al., 2019) remains controversial and has yet to be reproduced. Therefore, droplets resembling a mixture of B and T cell CITE-seq or VDJ-seq profiles might be enriched in doublets and/or multiplets.

We therefore propose a computational cell doublet/multiplet detection approach, MLtiplet, that leverages the additional granularity of multimodal scRNA-seq experiments using CITE-seq and immune receptor profiling, respectively. This approach identified droplets that correspond to mixed-cell droplet profiles, including those with mutually exclusive CITE-seq profiles or multiple TCR/BCR profiles. As these CITE-seq or TCR/BCR profiles only identify a subset of *possible doublets and/or multiplets*, we subsequently applied a generalized linear model to fit the profile of these *true doublets and/or multiplets* compared with the remainder of the droplets. The model was then used as a classifier to detect doublets and/or multiplets for which the CITE-seq or VDJ-seq data were not available or appropriate. This approach incorporates features of the transcriptomic profiles into a single model that statistically distinguishes doublets and/or multiplets from true singlets. These transcriptional features include the relative number of mRNA molecules (nUMIs), and apoptosis-associated gene signatures. We show that doublets and/or multiplets identified from both CITE-seq and VDJ-seq data might be used as a combined training dataset for MLtiplet for highest sensitivity and specificity.

RESULTS

Here, we propose a model-based classification of doublets based on the profiles of identified mixed-cell droplets (Figures 1A and S1). We applied these approaches to publicly available datasets containing RNA-seq, CITE-seq, and VDJ-seq modalities of peripheral blood mononuclear cells (PBMCs) from three healthy individuals (https://support.10xgenomics.com/single-cell-vdj/datasets). These were pre-processed according to the

Figure 1. Multi-omics aids the identification of doublets and/or multiplets

(A) Schematic of approach to identify scRNA-seq doublets and/or multiplets using the CITE-seq and VDJ modalities. Droplets with a transcriptome resembling non-B or non-T cells that captured BCR or TCR sequences, respectively, were considered as potential doublets and/or multiplets.

(B) Uniform manifold approximation and projection (UMAP) dimensionality reduction of three healthy PBMC datasets colored by cell type.

(C) Examples of the CITE-seq levels between CD3, CD4, and CD8 for the three individuals for the B cell cluster, with the red lines corresponding to the CITE-seq positivity thresholds.

(D) UMAP dimensionality reduction of three healthy PBMCs colored by VDJ doublets (left, co-capture of discordant VDJs) or CITE-seq doublets (right, co-capture of the corresponding mutually exclusive CITE-seq pair). Homotypic doublets were defined as those containing multiple BCRs or multiple TCRs, and the heterotypic doublets are defined as the remainder (droplets containing both BCR(s) and TCR(s) or droplets containing BCRs or TCRs that do not have transcriptional profiles that resemble B or T cells, respectively).

(E) Generalized additive models fitted on percentage mitochondrial genes versus percentage ribosomal genes, R^2 values shown top right; from the HEK293 dataset.

(F) Mito-ribo ratio values per enriched HEK293 populations. p values were calculated across groups by using Wilcoxon test with Bonferroni correction for pairwise comparisons.

(G) The relative numbers of genes (nGenes), number of RNA molecules (nUMI), mito-ribo ratio (mitoribo_ratio), and nUMIs_VDJ per droplet for the VDJ-identified doublets and/or multiplets (left) and the CITE-seq identified doublets and/or multiplets (right). "Other" refers to droplets that were not identified as doublets and/or multiplets from the VDJ-seq or CITE-seq data. The p values of the differences between the feature distributions of the doublet/multiplets detected and the remainder of the droplets provided (two-sided Wilcoxon test). ****p < 0.00005 (Wilcoxon test). Abbreviation is as follows: CLR, centered log-ratio transformed.

Seurat analysis pipeline to exclude low-quality droplets (with low numbers of captured genes and RNA molecules) and were batch corrected through *harmony* (see the STAR Methods). A total of 26,080 droplets were retained after filtering (7,024–11,382 per sample), for which the broad immune cell types were annotated through differential gene expression and CITE-seq marker expression (Figures 1B and S1C).

The first step for doublet/multiplet detection is to identify mixed-cell droplet profiles that can be deduced from the data. This might be achieved through the leveraging of CITE-seq and immune receptor profiling. The CITE-seq approach allows for the potential identification of cell multiplets through the occurrence of co-staining for cell-type-exclusive canonical markers that are not expected to be co-expressed. For example, the majority of B cells identified from the scRNA-seq gene expression do not have high CITE-seq levels for the T cell markers CD3, CD4, or CD8 (Figures 1C, S1D, and S1E). However, of the B cells that have high CITE-seq levels for these T cell markers, the CITE-seq patterns reflect those expected for hybrid of B cells and $CD4^+$ or $CD8^+$ T cells: high CITE-seq levels of CD3 and CD4 or CD3 and CD8 together, but not CD4 and CD8 together. This is suggestive of true doublets reflecting a mixture of CITE-seq patterns that might be used to infer doublets and/or multiplets. From 26,080 filtered cells after quality control, 2,068 droplets were identified with CITE-seq profiles resembling mixed-cell-type doublet/multiplet droplets (Table S1). Each cell-type-specific mutually exclusive CITE-seq pair is observed in different UMAP clusters (Figures 1D and S2A), with B-T cell hybrids ($CD19^+CD4^+$, $CD19^+CD8^+$, and $CD19^+CD3^+$ droplets) observed primarily in the "B cell" cluster, and B-cell-myeloid hybrids ($CD19^+CD16^+$) and T cell-neutrophil/monocyte hybrids ($CD4^+$ $CD16^+$) observed primarily in the "neutrophils/monocyte" cluster.

Similarly, the VDJ-seq approach allows for the potential identification of doublets and/or multiplets through the occurrence of droplet co-capture of multiple functional BCR heavy or light chains (suggestive of two B cells captured), multiple functional TCR alpha or beta chains (suggestive of two T cells captured), or combinations of BCRs and TCRs (suggestive of a B cell and a T cell captured). In addition, droplets with a transcriptome resembling non-B or non-T cells that captured BCR or TCR sequences, respectively, were considered as potential doublets and/or multiplets. Given the common dual TCR alpha chain expression, these droplets were not included as markers of potential doublets and/or multiplets. From 26,080 filtered cells after QC, 835 droplets were identified with VDJ-seq profiles resembling doublet/multiplet droplets (Table S1), and are enriched in the B cell and T cell clusters (Figures 1D and S2B).

As these CITE-seq or TCR/BCR profiles will only identify a subset of *possible doublets and/or multiplets*, the second step is to then apply a model to fit the profile of these *identified doublets and/or multiplets* to be used as a classifier to predict doublets and/or multiplets in the remaining cells, such as for those where the CITE-seq or VDJ-seq was not available/appropriate. This approach might incorporate any single-cell cellular, transcriptional, CITE-seq, or VDJ-seq variable into a single model that statistically distinguishes doublets and/or multiplets from true singlets, such as the relative number of captured genes and RNA molecules per droplet that are expected to be elevated in droplets that captured more than one cell.

We also consider a transcriptional marker for the identification of low-quality or dying cells in scRNA-seq data given the propensity of these cells to aggregate (Cui et al., 2016; Kuonen et al., 2010), thus potentially providing a useful marker for dying/low-quality cells that are more prone to forming doublet/multiplet aggregates. Current computational identification of low-quality cells relies on arbitrary thresholds of percentage representation of mitochondrial gene counts (Ilicic et al., 2016); however, such thresholds have been shown to perform poorly on ground truth datasets (Ordonez-Rueda et al., 2020). We hypothesized that downregulation of RNA encoding ribosomal proteins (rRNA), associated with cellular stress (Albert et al., 2019) and apoptosis (Lin et al., 1994), is a technical droplet feature that might improve sensitivity for detection of apoptotic or pre-apoptotic cells. Indeed, principal-component analysis on a ground truth scRNA-seq dataset of FACS sorted apoptotic HEK cells (Ordonez-Rueda et al., 2020) revealed that the main variables driving dead cell identification were low percentage ribosomal genes and high percentage mitochondrial genes; representing 49.7% of all variance explained by the first principle component (Figures 1E and S2C–S2E). Therefore, we created an additional variable to be included in the doublet detection model, named the mito-ribo ratio, calculated by the proportion of mitochondrial RNA (mtRNA) divided by the sum of the proportion of rRNA and mtRNA. Indeed, the mito-ribo ratio was elevated in apoptotic cells (Figure 1F). By thresholding on the local minimum (0.47) of a fitted Gaussian mixture model (GMM), we were able to discriminate 84% of late apoptotic cells with >1,537 detected genes, threshold for doublets identified by a GMM (traditional arbitrary filter is >2,500 genes), and observed a trend toward higher scores in early apoptotic cells (Figure 1F).

We next tested whether these features might discriminate the identified doublets and/or multiplets from the remainder of the droplets (which should be enriched for singlets). As expected, the relative number of genes (nGenes) and mRNA molecules (nUMIs) detected was significantly higher in the identified doublets and/or multiplets through the VDJ-seq and CITE-seq approaches than in the remainder of droplets ($p < 1e{-10}$, Figure 1G). The mito-ribo ratio varied significantly between droplet types and by cell cycle (Figure S2F) ($p < 1e{-10}$, ANCOVA using sample as an additional covariate), which was consistent across independent samples. However, the mito-ribo ratio was significantly elevated in droplets identified as doublets and/or multiplets (Figure 1G, $p < 1e{-10}$), supporting this as an informative metric to identify doublets and/or multiplets in combination with other droplet features.

We then applied a logistic regression by using the generalized linear model to fit the profile of these *identified doublets and/or multiplets* compared with the remainder of the droplets (enriched for *true singlets*), using the mito-ribo ratio, the per-sample centred log-ratio transformed nUMI counts and the module scores for each cell type or cluster as model inputs (Figure S1A). The module score is a per-cell score representing the relative likelihood of a cell being a member of a particular cell type/cluster (using the Seurat *AddModuleScore* function of the top 5 differentially expressed genes for each

CellPress
OPEN ACCESS

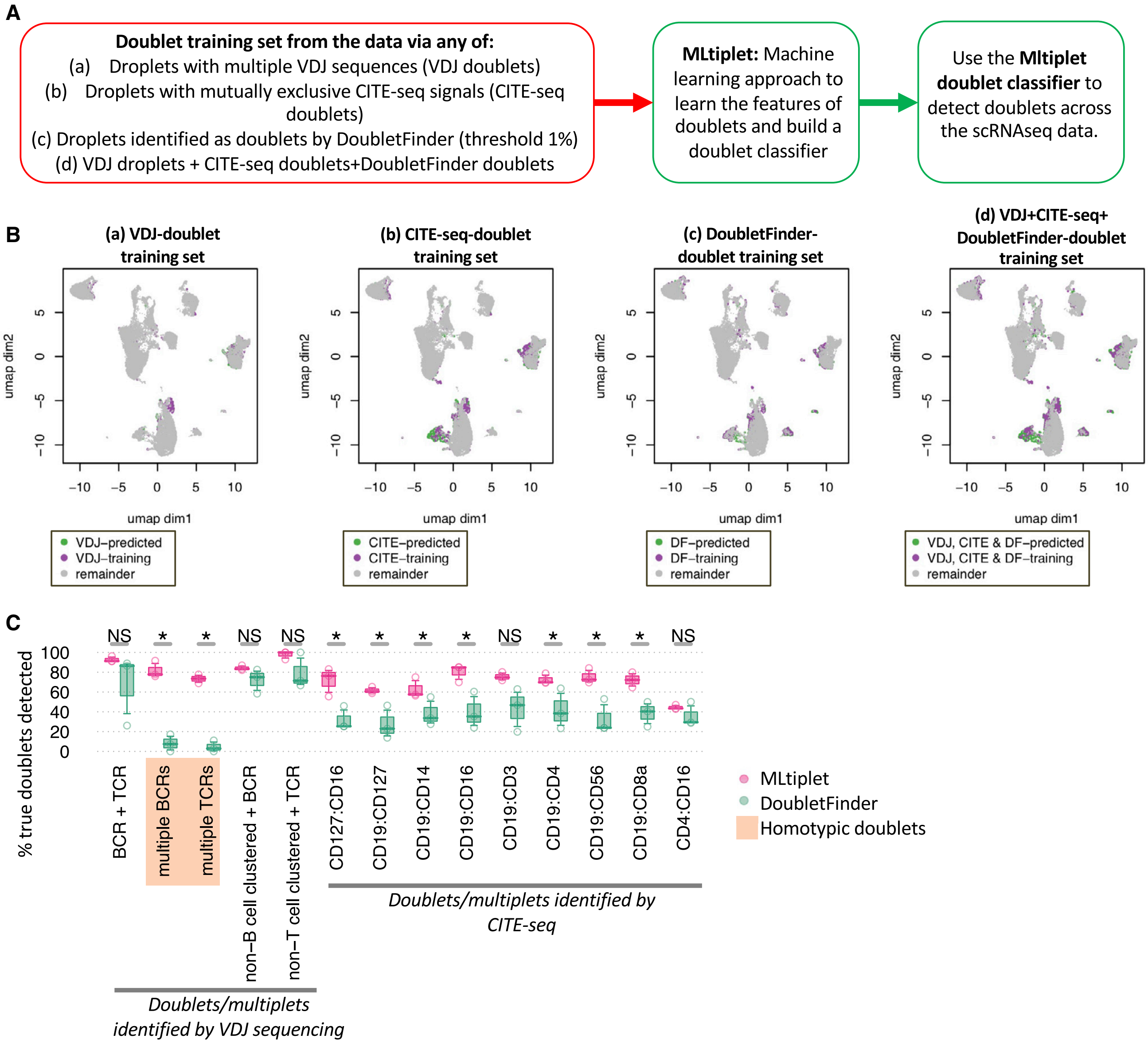

Figure 2. Machine learning applied to doublets and/or multiplet training data captures both homotypic and heterotypic doublets and/or multiplet

(A) Schematic of the MLtuplet applied to the healthy PBMC data.

(B) UMAP dimensionality reductions of three healthy PBMCs colored by MLtiplet-predicted singlets and training and predicted doublets across the VDJ-seq, CITE-seq, DoubletFinder training sets, and a training set combining all three approaches.

(C) The proportion of VDJ and CITE-seq identified (true) doublets and/or multiplets that were identified as doublets by MLtiplet and DoubletFinder, grouped by doublet type. The doublets highlighted in orange are homotypic doublets (comprised of multiple cells of similar transcriptional types, namely B or T cells). *$p < 0.05$ (two-way Wilcox test, corrected for multiple testing). p values are calculated between the MLtiplet-predicted doublets and/or multiplets and singlets by using two-sided Wilcoxon test, and *$p < 0.05$.

cell type/cluster). This provides the model with parameters associated with cell-type mixing as a result of hybrid transcriptomes present in droplets containing more than one cell. This model was then used as a classifier to detect the remaining doublets and/or multiplets for which the CITE-seq and/or VDJ-seq was not available/appropriate (Figure S1A), named MLtiplet. This model approach allows for the input of different training doublet/multiplet datasets, depending on the data available (Figures 2A and 2B). This provides a doublet/multiplet probability for each droplet based on their droplet features (Figures S2H, S3A, and S3B). MLtiplet was able to identify 791, 2,332, and 2,283 doublets and/or multiplets in the healthy PBMCs by using the VDJ-seq, CITE-seq, and DF training sets, respectively, primarily in the "B cell CD5^{+}GZMB^{-}

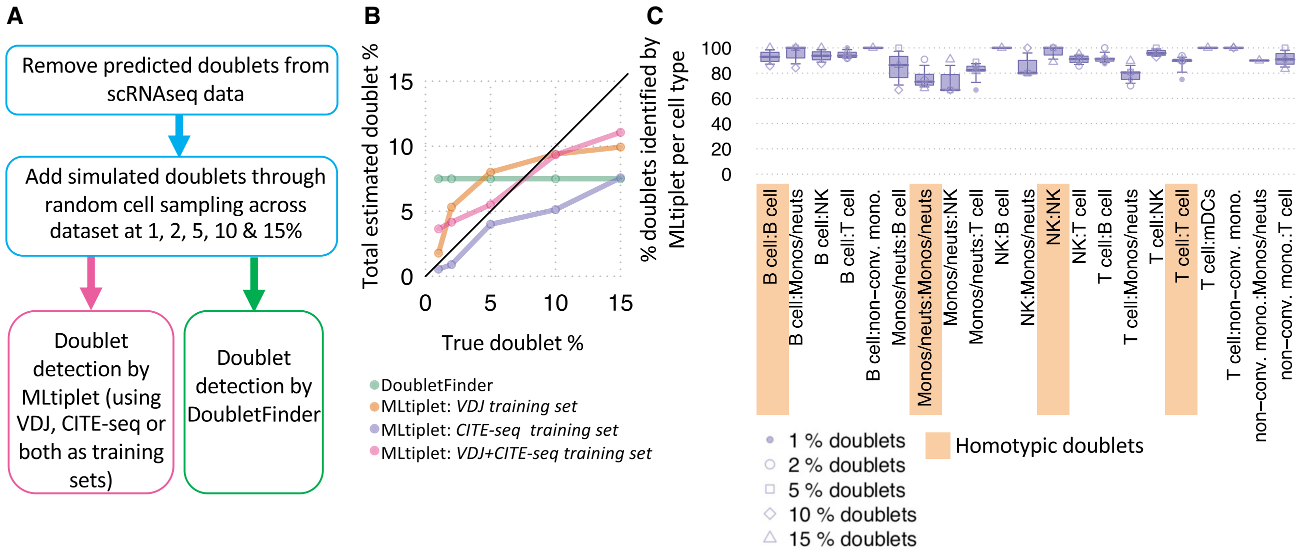

Figure 3. MLtiplet is both sensitive and specific on simulated doublets datasets, and scales with doublet proportion

(A) Schematic of the comparison of the doublet detection methods using simulated data.

(B) The estimated proportion of doublets across the simulated datasets using either DoubletFinder or the classifier based on VDJ-identified doublets, CITE-seq identified doublets, or both. The black line corresponds to y = x.

(C) The percentages of doublets identified by MLtiplet per cell type across the different simulated datasets. The point shapes correspond to the simulated dataset for which the percentage of true doublets was either 1%, 2%, 5%, 10%, or 15%. The homotypic doublets are highlighted in orange.

memory," "B cell CD5$^-$GZMB$^+$CD27$^-$," and "unconventional monocyte" clusters (Table S1).

Assuming that the *identified doublets and/or multiplets* are enriched for true doublets and/or multiplets, then the sensitivity of doublet detection might be quantified through the proportion of correctly labeled *identified doublets and/or multiplets* (Figure 2A). The sensitivity of doublet/multiplet detection of MLtiplet was significantly higher than the established DoubletFinder method (using default parameters, Figure 2C), both for heterotypic doublets and/or multiplets (such as those identified from CITE-seq or TCR-BCR discordance) and homotypic doublets and/or multiplets (such as those with multiple BCRs or TCRs). Indeed, DoubletFinder was only able to identify <20% of homotypic doublets and/or multiplets with multiple BCRs or TCRs, compared with >70% using MLtiplet. The droplet features of the predicted doublets and/or multiplets using MLtiplet have elevated numbers of nUMIs and mito-ribo ratios (Figures S3B–S3D). Furthermore, the differentially expressed genes between droplets predicted to be doublets and/or multiplets compared with those predicted to be singlets per cluster revealed signals of mixed-cell populations (Figures S3E and S3F), such as elevated CD3E and CD3D in the doublets that clustered with the B cells, TRBC2 CD69 and MS4A1 in the doublets that clustered with the monocytes/neutrophils.

In general, the larger the training dataset the greater statistical power for pattern recognition for doublet detection (Vabalas et al., 2019). Therefore, we determined the effect of different types and sizes of doublet/multiplet training datasets on the doublet prediction. Decreasing the number of droplets per training dataset also reduced the proportion of doublets and/or multiplets classified. However, using only 139 CITE-seq identified doublets and/or multiplets (comprising only 20% of VDJ-seq identified doublets and/or multiplets, Table S2) as the training set resulted in 435 droplets classified as doublets and/or multiplets (36% of that from the full VDJ-seq training set). Only including VDJ- and CITE-seq-identified doublets and/or multiplets from a single sample in the training set resulted in the prediction of, on average, 41% of droplets classified as doublets and/or multiplets compared with using total corresponding training set across all samples. This suggests that datasets without the VDJ-seq and/or CITE-seq modalities datasets can be combined with datasets from which some doublets and/or multiplets can be identified to predict the majority of doublets and/or multiplets through machine learning of the droplet features.

Given that many currently used doublet detection methods require the prior estimation of multiplet rate, which is unknown in most scRNA-seq experiments, we compared the performance of MLtiplet and DoubletFinder on simulated data to assess how the doublet detection varies with true doublet proportions. Simulated RNA-seq, CITE-seq, and VDJ-seq data were generated to contain 1%, 2%, 5%, 10%, or 15% doublets, containing both heterotypic and homotypic doublets (see the STAR Methods, Figure 3A). Indeed, using the default parameters for DoubletFinder (with a prior of 7.5% doublets), the estimated doublet proportion remained constant irrespective of the true doublet proportion (Figure 3B). However, using either the VDJ-seq-identified doublets, CITE-seq-identified doublets, or both, as the training sets for MLtiplet, the estimated proportion of doublets scaled with the true proportion, with combined "VDJ-seq- and CITE-seq-identified doublets training set" performing best. Furthermore, MLtiplet accurately detected both heterotypic

CellPress
OPEN ACCESS

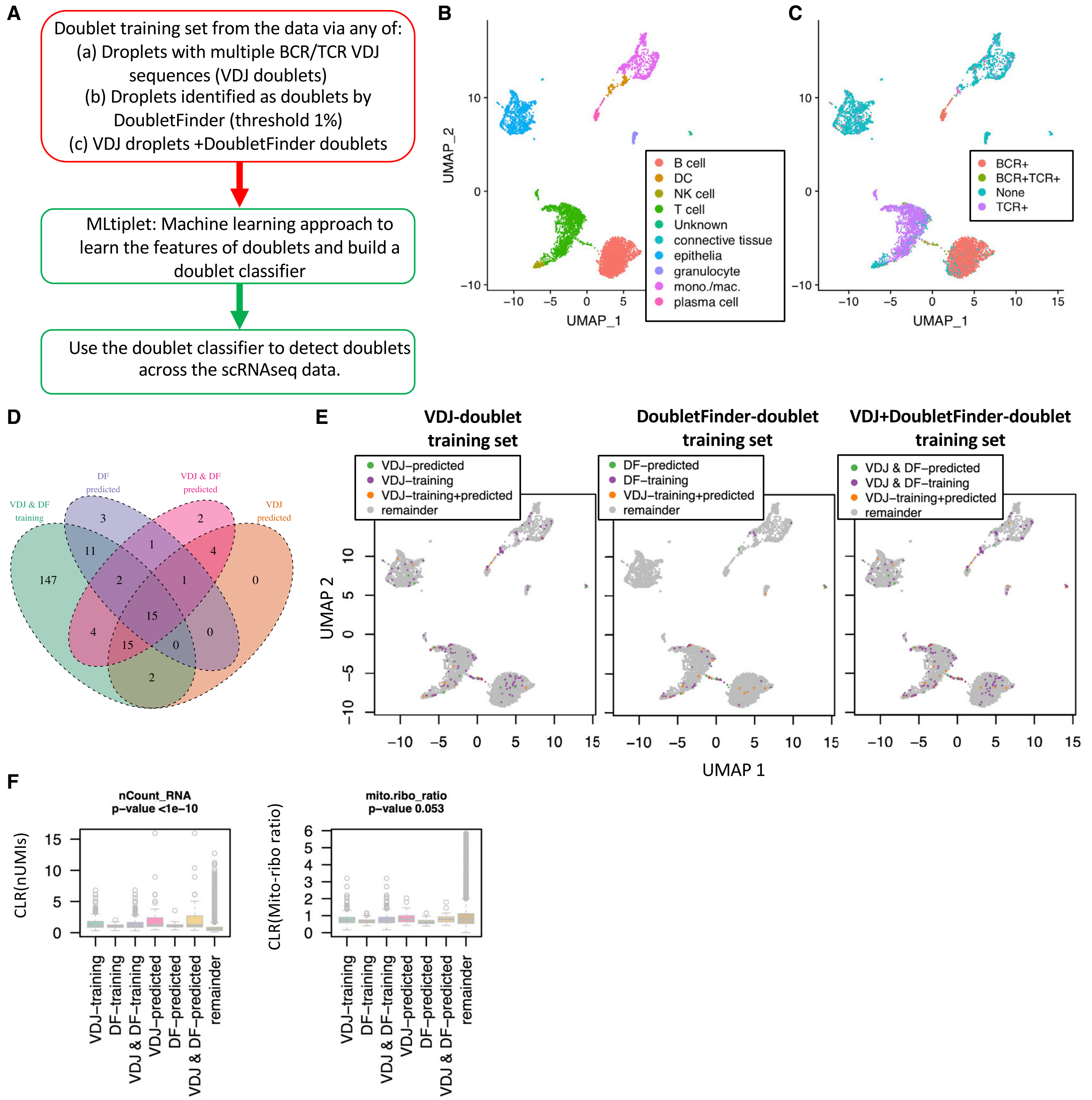

Figure 4. Validation of MLtiplet on an NSCLC tumor dataset

Doublet detection on a non-small cell lung cancer (NSCLC) dataset.

(A–C) Shown are the (A) schematic of the training datasets for doublet/multiplet prediction by MLtiplet. UMAP plots of (B) the annotated cell types and (C) VDJ-seq heterotypic doublets.

(D) Venn diagram showing the numbers of droplets used as the combined identified doublets and/or multiples using both DoubletFinder and VDJ-seq (green), and the predicted doublets and/or multiplets from MLtiplet using the DoubletFinder-derived training dataset (blue), VDJ-seq-derived training dataset (orange), and DoubletFinder plus VDJ-seq-derived training dataset (pink).

(E) UMAP plots of the training and predicted doublets and/or multiples using each approach.

(F) The relative numbers of RNA molecules (nUMI) and mito-ribo ratio (mitoribo_ratio) per cell for the VDJ-identified doublets and/or multiplets, CITE-seq-identified doublets and/or multiplets, MLtiplet-predicted doublets and/or multiplets, and the remainder (predicted singlets by MLtiplet).

and homotypic doublets at a rate of >68% across all doublet combinations (Figure 3C). Therefore, we show that VDJ-seq- and CITE-seq-identified doublets might be used individually or in tandem for scalable detection of doublets and/or multiplets without prior knowledge of doublet proportions, and these methods outperform current doublet detection methods.

We next tested this approach on a sample derived from solid tissue, namely from a non-small cell lung cancer dataset (https://support.10xgenomics.com/single-cell-vdj/datasets, Figure 4). Here, we compare different input training doublet datasets for MLtiplet using VDJ-seq-identified doublets and/or multiplets, DoubletFinder-identified doublets and/or multiplets, or a combination of the two (Figure 3A). The three approaches identified similar distributions of doublets and/or multiples, with the combined input training datasets resulting in the highest number of predicted doublets (Figure 3B). Although we show that the number of doublets and/or multiplets identified in the B and T cell clusters are lower using DoubletFinder than using VDJ-seq (Table S3), running these doublet and/or multiplet training datasets through MLtiplet resulted in comparable numbers of predicted doublets and/or multiplets in each cell-type cluster. Differential gene expression analysis between the predicted doublets and/or multiplets compared with the predicted singlets per droplet cluster (Figure S3G) confirmed the mixed transcriptomic profiles of the predicted doublets and/or multiplets. This is exemplified by the elevated CD3D and CD3E expression in predicted doublets and/or multiplets within the "B cell", “DC,” and “plasma cell” clusters, which is indicative of T cell contamination. This demonstrates that the input training datasets for MLtiplet might be broadly generalizable to other inputs when VDJ-seq or CITE-seq are not be available. We further demonstrate this approach on a murine dataset derived from the PBMCs from two mouse strains (BALB/c and C57BL/6, https://support.10xgenomics.com/single-cell-vdj/datasets) (Figures S4A–S4F).

We also observed an enrichment of doublets and/or multiplets in specific cell clusters: (1) the majority of the “non-conventional monocyte” cluster were identified as doublets and/or multiplets in the healthy PBMC dataset (Figure 1D), suggesting that this cluster predominantly represents a subset of related hybrid transcriptomes. (2) Both homotypic doublets (containing >1 BCR chain type) and heterotypic doublets (containing BCRs and TCRs) were observed in the B cell cluster, suggesting that B-T cell hybrid transcriptome co-cluster with the true singlet B cell transcriptome populations. It is noted that there is an enrichment of some IGHV genes within the doublet droplets, suggesting that these cell-cell doublets are potentially as a result of cell-cell interactions that are enriched for certain antigen specificities (Figure S4G).

DISCUSSION

Overall, we demonstrate a doublet/multiplex droplet detection approach by using machine learning to predict doublets based on the features associated with identified hybrid droplets that can be applied to scRNA-seq datasets containing only VDJ-seq or CITE-seq information, or both. In theory, this might also be applied to scRNA-seq-only datasets through the identification of gene expression profiles that resemble hybrid transcriptomes, such as those identified through Scrublet or DoubletFinder to then feed into the doublet prediction model. The deficiency of previous doublet prediction methods for identifying homotypic doublets and/or multiplets is highlighted here by the CITE-seq and VDJ-seq analyses, and it is recommended to leverage these multi-omic single-cell data types for the generation of high-quality datasets. Thus, this presents a powerful approach, particularly for inflammatory cell-dominant scRNA-seq samples, to ensure high-quality scRNA-seq minimizing false discoveries of rare cell types from both homotypic and heterotypic doublets and/or multiplets or differential gene expression signatures, and finally ensuring the reproducibility of biological findings.

Limitations of the study

Although identification of unseen homotypic and heterotypic cell multiplets using MLtiplet appears both sensitive and specific in our hands, the *a priori* assumptions do pose some limitations. Firstly, the definitive validation of mutually exclusive markers in the literature remains in evolution. Modeling multimodal cell doublets and/or multiplets, first required the curation of mutually exclusive CITE-seq markers and VDJ-seq chains based on best evidence to date (CD19 + CD3, CD19 + CD4, CD19 + CD8a, CD19 + CD56, CD19 + CD16, CD19 + CD14, CD19 + CD127, CD19 + CD56, CD4 + CD16, CD127 + CD16, STAR Methods). However, as the granularity of single-cell characterization improves, novel bona fide mixed-phenotypes arising from cellular plasticity or invariant VDJ-seq allelic inclusion might be revealed. To counter this limitation, we designed MLtiplet to allow the user to make heuristic decisions regarding which mutually exclusive markers to feed into the model, thus futureproofing for novel discoveries. Secondly, biological differences that might affect the absolute number of transcripts per cell, such as the physical cell size and proliferation status, cannot be modeled reliably. The use of nUMIs counts assumes a degree of homogeneity across these variables at the true single-cell level; therefore, physically large cells that are comparable in size to true multiplets might be incorrectly classified as doublets. Indeed, this is also a limitation for traditional exclusion of potential multiplets or poor-quality cells by thresholding on nUMIs, therefore it remains an unaddressed issue of cell size identification at the pre-bioinformatic level. Thirdly, due to the predominant use or commercial availability of CITE-seq antibodies targeting inflammatory cell markers, we have only been able to benchmark MLtiplet for identification of cell multiplets within these limitations. However, in theory, MLtiplet might take any custom barcode-conjugated antibodies targeting cell-surface markers that are mutually exclusive between two or more cell types. Finally, our proposed method’s performance scales proportionally to the breadth of multi-omic data available. Although single-cell experiments are becoming more cost-effective, they remain prohibitively expensive with the addition of other modalities. Where mutually exclusive markers and/or VDJ chains are not present in the dataset, the sensitivity and specificity of the model reduces and thus this is a limitation that is proportional to the available resources at hand.

STAR★METHODS

Detailed methods are provided in the online version of this paper and include the following:

- KEY RESOURCES TABLE
- RESOURCE AVAILABILITY
  - Lead contact
  - Materials availability
  - Data and code availability
- METHOD DETAILS
  - scRNA-seq pre-processing and batch correction
  - CITE-seq-identified doublet/multiplet training set
  - VDJ-seq-identified doublet/multiplet training set
  - Mito-ribo ratio
  - MLtiplet model fitting
  - Simulated data containing known proportions of doublets
- QUANTIFICATION AND STATISTICAL ANALYSES

SUPPLEMENTAL INFORMATION

Supplemental information can be found online at https://doi.org/10.1016/j.crmeth.2021.100008.

ACKNOWLEDGMENTS

We would like to thank Dr Santiago Revale for advice for the scRNA-seq processing pipeline. R.J.M.B.-R., L.E.O., and F.A.T. were supported by the Wellcome Trust. B.S. was supported by the Association of British Neurologists via the Patrick Berthoud Charitable Trust. E.B.E. would like to acknowledge G.C. Bugachi and CONACyT (Mexico) for the financial support during his postgraduate studies. This work was supported by the Wellcome Trust. No ethical approval was required for this study.

AUTHOR CONTRIBUTIONS

R.J.M.B.-R. and B.S. planned the study. R.J.M.B.-R., B.S., E.B.-E., and C.E.W. analyzed the data. All authors provided intellectual contributions to analyses and interpretation. R.J.M.B.-R., B.S., and E.B.-E. wrote the manuscript. All authors edited the manuscript.

DECLARATION OF INTERESTS

R.J.M.B.-R. is a co-founder of Alchemab Therapeutics Ltd and consultant for Alchemab Therapeutics Ltd and GSK. F.A.T. is a consultant for Alchemab Therapeutics Ltd.

Received: September 25, 2020
Revised: March 9, 2021
Accepted: March 26, 2021
Published: May 12, 2021

REFERENCES

Ahmed, R., Omidian, Z., Giwa, A., Cornwell, B., Majety, N., Bell, D.R., Lee, S., Zhang, H., Michels, A., Desiderio, S., et al. (2019). A public BCR present in a unique dual-receptor-expressing lymphocyte from type 1 diabetes patients encodes a potent T cell autoantigen. Cell *177*, 1583–1599.e16.

Albert, B., Kos-Braun, I.C., Henras, A.K., Dez, C., Rueda, M.P., Zhang, X., Gadal, O., Kos, M., and Shore, D. (2019). A ribosome assembly stress response regulates transcription to maintain proteome homeostasis. eLife *8*, e45002.

Azizi, E., Carr, A.J., Plitas, G., Cornish, A.E., Konopacki, C., Prabhakaran, S., Nainys, J., Wu, K., Kiseliovas, V., Setty, M., et al. (2018). Single-cell map of diverse immune phenotypes in the breast tumor microenvironment. Cell *174*, 1293–1308.e36.

Bais, A.S., and Kostka, D. (2020). scds: computational annotation of doublets in single-cell RNA sequencing data. Bioinformatics *36*, 1150–1158.

Barreto, V., and Cumano, A. (2000). Frequency and characterization of phenotypic Ig heavy chain allelically included IgM-expressing B cells in mice. J. Immunol. *164*, 893–899.

Casellas, R., Zhang, Q., Zheng, N.Y., Mathias, M.D., Smith, K., and Wilson, P.C. (2007). Igkappa allelic inclusion is a consequence of receptor editing. J. Exp. Med. *204*, 153–160.

Cui, L.L., Kinnunen, T., Boltze, J., Nystedt, J., and Jolkkonen, J. (2016). Clumping and viability of bone marrow derived mesenchymal stromal cells under different preparation procedures: a flow cytometry-based in vitro study. Stem Cells Int. *2016*, 1764938.

Depasquale, E.A.K., Schnell, D.J., Van Camp, P.J., Valiente-Alandi, I., Blaxall, B.C., Grimes, H.L., Singh, H., and Salomonis, N. (2019). DoubletDecon: deconvoluting doublets from single-cell RNA-sequencing data. Cell Rep. *29*, 1718–1727 e8.

Ilicic, T., Kim, J.K., Kolodziejczyk, A.A., Bagger, F.O., McCarthy, D.J., Marioni, J.C., and Teichmann, S.A. (2016). Classification of low quality cells from single-cell RNA-seq data. Genome Biol. *17*, 29.

Kang, H.M., Subramaniam, M., Targ, S., Nguyen, M., Maliskova, L., McCarthy, E., Wan, E., Wong, S., Byrnes, L., Lanata, C.M., et al. (2018). Multiplexed droplet single-cell RNA-sequencing using natural genetic variation. Nat. Biotechnol. *36*, 89–94.

Korsunsky, I., Fan, J., Slowikowski, K., Zhang, F., Wei, K., Baglaenko, Y., Brenner, M., Loh, P.-R., and Raychaudhuri, S. (2018). Fast, sensitive, and flexible integration of single cell data with Harmony. Nat. Methods *16*, 1289–1296.

Kuonen, F., Touvrey, C., Laurent, J., and Ruegg, C. (2010). Fc block treatment, dead cells exclusion, and cell aggregates discrimination concur to prevent phenotypical artifacts in the analysis of subpopulations of tumor-infiltrating CD11b(+) myelomonocytic cells. Cytometry A *77*, 1082–1090.

Lin, C.H., Palma, J.F., and Solomon, W.B. (1994). Phorbol ester induction of differentiation and apoptosis in the K562 cell line is accompanied by marked decreases in the stability of globin mRNAs and decreases in the steady state level of mRNAs encoding for ribosomal proteins L35, L31, L27, and L21. Cell Mol. Biol. Res. *40*, 13–26.

McGinnis, C.S., Murrow, L.M., and Gartner, Z.J. (2019). DoubletFinder: doublet detection in single-cell RNA sequencing data using artificial nearest neighbors. Cell Syst. *8*, 329–337.e4.

Ordonez-Rueda, D., Baying, B., Pavlinic, D., Alessandri, L., Yeboah, Y., Landry, J.J.M., Calogero, R., Benes, V., and Paulsen, M. (2020). Apoptotic cell exclusion and bias-free single-cell selection are important quality control requirements for successful single-cell sequencing applications. Cytometry A *97*, 156–167.

Padovan, E., Casorati, G., Dellabona, P., Meyer, S., Brockhaus, M., and Lanzavecchia, A. (1993). Expression of two T cell receptor alpha chains: dual receptor T cells. Science *262*, 422–424.

Stegle, O., Teichmann, S.A., and Marioni, J.C. (2015). Computational and analytical challenges in single-cell transcriptomics. Nat. Rev. Genet. *16*, 133–145.

Stoeckius, M., Hafemeister, C., Stephenson, W., Houck-Loomis, B., Chattopadhyay, P.K., Swerdlow, H., Satija, R., and Smibert, P. (2017). Simultaneous epitope and transcriptome measurement in single cells. Nat. Methods *14*, 865–868.

Stoeckius, M., Zheng, S., Houck-Loomis, B., Hao, S., Yeung, B.Z., Mauck, W.M., 3rd, Smibert, P., and Satija, R. (2018). Cell Hashing with barcoded

antibodies enables multiplexing and doublet detection for single cell genomics. Genome Biol. *19*, 224.

Vabalas, A., Gowen, E., Poliakoff, E., and Casson, A.J. (2019). Machine learning algorithm validation with a limited sample size. PLoS One *14*, e0224365.

Wolock, S.L., Lopez, R., and Klein, A.M. (2019). Scrublet: computational identification of cell doublets in single-cell transcriptomic data. Cell Syst. *8*, 281–291.e9.

Zheng, G.X., Terry, J.M., Belgrader, P., Ryvkin, P., Bent, Z.W., Wilson, R., Ziraldo, S.B., Wheeler, T.D., Mcdermott, G.P., Zhu, J., et al. (2017). Massively parallel digital transcriptional profiling of single cells. Nat. Commun. *8*, 14049.

CellPress
OPEN ACCESS

STAR★METHODS

KEY RESOURCES TABLE

REAGENT or RESOURCE	SOURCE	IDENTIFIER
Deposited data		
10X 5′ GEX + VDJ-seq of healthy human PBMCs	https://support.10xgenomics.com/single-cell-vdj/datasets	vdj_v1_hs_pbmc
10X 5′ GEX + VDJ-seq of healthy human PBMCs	https://support.10xgenomics.com/single-cell-vdj/datasets	vdj_v1_hs_pbmc2
10X 5′ GEX + VDJ-seq of healthy human PBMCs	https://support.10xgenomics.com/single-cell-vdj/datasets	vdj_v1_hs_pbmc3
10X 5′ GEX + VDJ-seq of human NSCLC sample	https://support.10xgenomics.com/single-cell-vdj/datasets	vdj_v1_hs_nsclc
10X 5′ GEX + VDJ-seq of healthy mouse PBMCs	https://support.10xgenomics.com/single-cell-vdj/datasets	vdj_v1_mm_c57bl6_pbmc
10X 5′ GEX + VDJ-seq of healthy mouse PBMCs	https://support.10xgenomics.com/single-cell-vdj/datasets	vdj_v1_mm_balbc_pbmc
Software and algorithms		
DoubletFinder	https://www.sciencedirect.com/science/article/pii/S2405471219300730#sec4	https://github.com/chris-mcginnis-ucsf/DoubletFinder
Seurat	https://www.nature.com/articles/nbt.3192	https://github.com/satijalab/seurat
MLtiplet	This paper	https://github.com/rbr1/MLtiplet

RESOURCE AVAILABILITY

Lead contact

Further information and requests for resources and reagents should be directed to and will be fulfilled by the Lead Contact, Rachael Bashford-Rogers (rbr1@well.ox.ac.uk).

Materials availability

This study did not generate new unique reagents.

Data and code availability

The code generated during this study are available at https://github.com/Bashford-Rogers-lab/MLtiplet. Original scRNA-seq data for the paper is available from https://support.10xgenomics.com/single-cell-vdj/datasets.

METHOD DETAILS

scRNA-seq pre-processing and batch correction

The 10X Genomics' Chromium scRNA-seq output data from three healthy donors' PBMCs (https://support.10xgenomics.com/single-cell-vdj/datasets) was merged using the *Seurat* package in R. First, we filtered low-quality cells using Seurat (version 2.3.4), retaining cells with detected gene numbers >500, detected number of RNA molecules (>1000 unique molecular identifiers (UMIs) and <25% mitochondrial UMIs). We then normalised the gene counts for each cell using the *ScaleData* function in *Seurat* with the default parameters. Integration and batch correction were performed using the *harmony* package(Korsunsky et al., 2018) with default parameters, and using sample ID as a variable to regress out. The resulting harmony embeddings were used in the data visualized using a Uniform Manifold Approximation and Projection (UMAP) projection and subsequent clustering of cell types.

CITE-seq-identified doublet/multiplet training set

The raw CITE-seq data were normalised through centred log-ratio transformed (CLR) per sample (Figure S1B). Cells/droplets that were positive for each CITE-seq antibody were determined using the following method (Figures S1B and S2): for each CITE-seq antibody, the normalised CITE-seq levels between cell populations with high corresponding gene expression (such as T cells for CD3) and low corresponding gene expression (such as B cells and myeloid cells for CD3) were the input into a linear classifier (linear discriminant analysis, LDA). This was then used to determine the optimal threshold for distinguishing the threshold between

CITE-seq positive and CITE-seq negative cells/droplets. Each cell/droplet was then classified to determine whether they are positive or negative. This was performed for each CITE-seq antibody. Mutually exclusive CITE-seq markers for the healthy PBMC dataset was: CD19 + CD3, CD19 + CD4, CD19 + CD8a, CD19 + CD56, CD19 + CD16, CD19 + CD14, CD19 + CD127, CD19 + CD56, CD4 + CD16, CD127 + CD16. Downstream analyses were performed on only the CITE-seq probes with classification of corresponding gene expression positive cells with a sensitivity of >70%.

The training set using the CITE-seq identification therefore relies on the co-positivity of mutually exclusive cell marker antibodies (cell-type-specific mutually exclusive CITE-seq pairs). This mutually exclusive list can be determined through either a literature search or comparison of the gene expression/CITE-seq levels between cell subtypes.

VDJ-seq-identified doublet/multiplet training set

The raw VDJ sequencing was filtered to remove VDJ sequences with non-productive sequences and fewer than 6 UMIs. VDJ-seq-identified doublets/multiplets were defined as droplets containing either (a) a BCR chain (IGH and/or IGK/L) and a TCR chain (TRA and/or TRB), (b) multiple BCRs (2 or more IGH chains and/or 2 or more IGK/L chains), (c) multiple TCRs (2 or more TRA chains and/or 2 or more TRB chains), (d) droplets that do not co-cluster with B cells via gene expression and contain a BCR chain (IGH and/or IGK/L), and (e) droplets that do not co-cluster with T cells via gene expression and contain a TCR chain (TRA and/or TRB).

Mito-ribo ratio

As a discriminant of singlets from doublets/multiplets, the mito-ribo ratio was used as a covariate, denoted by:

$$mito.ribo \ \ ratio = \frac{\sum m}{\sum (m+r)}$$

Where m is the per cell mtRNA UMI percentage and r is the rRNA UMI percentage.

MLtiplet model fitting

A generalised linear model was used to fit the profile of these *identified doublets/multiplets* compared to the remainder of the droplets (enriched for *true singlets*), using the mito-ribo ratio, the per-sample CLR transformed nUMI counts and the module scores for each cell type as model inputs. The module score is a per-cell score representing the relative likelihood of a cell being a member of a particular cell type (using the Seurat *AddModuleScore* function of the top 5 differentially expressed genes for each cell type). This provides the model with parameters associated with cell-type mixing as a result of hybrid transcriptomes present in droplets containing more than one cell. First the *identified doublets/multiplets* and the remainder of the cells were each filtered to include only those within 2 standard deviations of the mean for each variable used (mito-ribo ratio and per-sample CLR transformed nGenes, nUMIs, and VDJ UMI counts). Then the generalised linear model was fitted in R using *glm* function using logistic regression, and used to predict doublets across the total dataset.

Simulated data containing known proportions of doublets

The doublet simulated data were based on the scRNA-seq, CITE-seq and VDJ dataset from the 3 healthy PBMC samples. First, the experimental data were "cleaned" to remove droplets with nUMI counts > median + interquartile range per sample. Then simulated doublets were introduced through random cell sampling and sum their gene expression, VDJ BCR UMI, TCR UMI and CITE-seq counts, and were classified with the combined original cell type annotation labels. 5 simulated datasets were generated that incorporated either 1, 2, 5, 10 or 15% simulated doublets. Each simulated dataset was then pre-processed as described in "scRNA-seq pre-processing and batch correction", and doublet detection methods were applied to the resulting datasets as described. MLtiplet accuracy of detected both heterotypic and homotypic doublets was calculated on all outputs for which there were >5 doublets.

QUANTIFICATION AND STATISTICAL ANALYSES

Statistical analyses were performed with R (https://www.r-project.org/). Data were presented as mean ± interquartile range. A p value of less than 0.05 was considered significant. * $p \leq 0.05$; ** $p \leq 0.005$, *** $p \leq 0.0005$. Statistical tests were performed via ANOVA for comparing differences in nUMIs, nGenes, or mito-ribo ratio between groups of cells. Heatmaps of gene expression and fold change between singlets and predicted doublets/multiplets were made with DoHeatmap in Seurat in R. Volcano plots of log p value against log fold change were done using ggplot2.

Cell Reports Methods, Volume *1*

Supplemental information

Double-jeopardy: scRNA-seq doublet/multiplet detection using multi-omic profiling

Bo Sun, Emmanuel Bugarin-Estrada, Lauren Elizabeth Overend, Catherine Elizabeth Walker, Felicia Anna Tucci, and Rachael Jennifer Mary Bashford-Rogers

Supplementary tables

Table S1. The numbers of doublets/multiplets doublets by VDJ-seq and CITE-seq per sample. Related to Figure 2.

		By sample			By gene expression type						
	**Doublet type**	**HC 1**	**HC 2**	**HC 3**	**B cells**	**Monocytes/ neutrophils**	**NK**	**T cells**	**mDCs**	**non-conv. monocytes**	**pDCs**
**Identified by VDJ-seq**	IGH+IGK/L+TRA or TRB	0	1	2	3	0	0	0	0	0	0
	3 of [IGH,IGK/L,TRA,TRB]	18	19	43	79	1	0	0	0	0	0
	IGH or IGK/L + TRA or TRB	22	23	54	95	1	0	3	0	0	0
	2x IGHs	54	12	29	93	2	0	0	0	0	0
	2x IGK/L	111	43	59	205	6	0	1	0	0	1
	2x TRBs	9	16	34	0	0	1	57	0	1	0
	2x IGH and 2x IGK/L	27	9	20	56	0	0	0	0	0	0
	2x TRAs and 2x TRBs	0	0	0	0	0	0	0	0	0	0
	non-B cell clustered + BCR	66	34	57	0	114	3	20	4	12	4
	non-T cell clustered + TCR	48	120	221	100	112	138	0	11	27	1
	**Total identified by VDJ-seq**	**253**	**210**	**372**	**333**	**225**	**141**	**77**	**15**	**39**	**5**
**Identified by CITE-seq**	CD127:CD16	95	118	283	16	110	262	26	1	81	0
	CD19:CD127	274	203	274	595	103	4	42	0	6	1
	CD19:CD14	92	40	71	16	162	3	13	1	6	2
	CD19:CD16	46	34	53	48	53	6	11	0	15	0
	CD19:CD3	124	91	170	285	51	3	44	0	1	1
	CD19:CD4	185	90	179	232	166	3	33	3	12	5
	CD19:CD56	38	46	51	64	46	6	17	0	2	0
	CD19:CD8a	62	40	72	91	46	4	31	0	2	0
	CD4:CD16	348	278	483	10	257	192	32	8	607	3
	**Total identified by CITE-seq**	**684**	**517**	**867**	**690**	**382**	**289**	**76**	**11**	**612**	**8**
**Identified by MLtiplet**	**VDJ-seq training set**	227	190	374	248	256	142	78	20	42	5
	**CITE-seq training set**	775	562	995	821	431	300	87	16	669	8
	**DF training set**	782	377	1124	393	585	173	222	358	398	154
	**Total droplets**	**7674**	**7024**	**11382**	**2982**	**5346**	**2461**	**13964**	**420**	**749**	**158**

Table S2. The number of predicted doublets using different doublet training sets for the healthy PBMC dataset. Related to Figure 2.

Training set	Number of droplets used in training set	Total doublets predicted	Total doublets predicted per sample			Total doublets predicted per cluster						
			**HC 1**	**HC2**	**HC3**	**B cells**	**Mono / neuts**	**NK**	**T cells**	**mDCs**	**non-conv. Mono.**	**pDCs**
**VDJ-training.0.2x**	139	435	142	105	188	289	82	10	5	3	40	6
**VDJ-training.0.4x**	278	640	209	165	266	423	123	16	18	28	29	3
**VDJ-training.0.6x**	416	843	263	218	362	535	167	48	36	22	27	8
**VDJ-training.0.8x**	555	1037	321	261	455	601	194	77	60	26	62	17
**VDJ-training.1x**	693	1219	371	309	539	736	221	98	73	27	53	11
**CITE-training.0.2x**	414	1891	595	455	841	1090	127	33	9	3	628	1
**CITE-training.0.4x**	828	2912	918	710	1284	1634	192	407	14	12	650	3
**CITE-training.0.6x**	1241	3671	1198	907	1566	2035	264	647	26	17	678	4
**CITE-training.0.8x**	1655	4079	1351	1035	1693	2385	328	634	32	17	679	4
**CITE-training.1x**	2068	4817	1561	1245	2011	2541	428	1083	56	21	684	4
**DoubletFinder-training.0.2x**	408	1374	453	318	603	260	175	6	3	257	521	152
**DoubletFinder-training.0.4x**	816	2021	723	456	842	551	361	87	10	308	553	151
**DoubletFinder-training.0.6x**	1224	2751	963	635	1153	889	518	261	24	331	575	153
**DoubletFinder-training.0.8x**	1632	3596	1245	853	1498	1143	678	626	41	342	611	155
**DoubletFinder-training.1x**	2040	4499	1521	1088	1890	1515	904	878	66	356	625	155
**VDJ & CITE-training.0.2x**	463	1772	551	415	806	943	150	10	5	18	642	4
**VDJ & CITE-training.0.4x**	926	2348	728	578	1042	1353	234	69	19	23	646	4
**VDJ & CITE-training.0.6x**	1389	3293	1069	812	1412	1885	356	289	29	61	663	10
**VDJ & CITE-training.0.8x**	1852	3947	1324	987	1636	2250	429	495	49	50	667	7
**VDJ & CITE-training.1x**	2314	4705	1559	1181	1965	2475	569	816	90	73	674	8
**VDJ, CITE & DoubletFinder-training.0.2x**	702	2222	755	512	955	784	288	134	11	240	613	152
**VDJ, CITE & DoubletFinder-training.0.4x**	1403	3844	1335	911	1598	1588	502	623	33	291	653	154
**VDJ, CITE & DoubletFinder-training.0.6x**	2104	5462	1799	1384	2279	2154	769	1313	76	326	670	154
**VDJ, CITE & DoubletFinder-training.0.8x**	2805	6589	2201	1689	2699	2603	1118	1554	131	343	686	154
**VDJ, CITE & DoubletFinder-training.1x**	3506	7453	2531	1896	3026	2788	1496	1772	187	365	691	154
**VDJ-training.HC_103**	139	570	325	96	149	433	114	2	7	2	11	1
**VDJ-training.HC_104**	278	439	120	125	194	219	83	41	21	15	56	4
**VDJ-training.HC_105**	416	596	113	177	306	302	113	76	30	21	32	22
**CITE-training.HC_103**	414	2283	877	511	895	1463	197	8	6	2	606	1
**CITE-training.HC_104**	828	2080	642	517	921	1127	162	99	16	13	658	5
**CITE-training.HC_105**	1241	3390	873	840	1677	1532	148	1002	39	9	655	5
**DoubletFinder-training.HC_103**	408	895	565	107	223	226	200	63	18	241	60	87
**DoubletFinder-training.HC_104**	816	1056	301	246	509	82	183	4	20	305	314	148
**DoubletFinder-training.HC_105**	1224	1667	353	452	862	456	365	32	25	297	341	151
**VDJ & CITE-training.HC_103**	463	2334	900	518	916	1479	227	6	5	2	614	1
**VDJ & CITE-training.HC_104**	926	1970	604	493	873	1018	176	71	18	29	654	4
**VDJ & CITE-training.HC_105**	1389	2746	758	661	1327	1243	214	570	37	25	649	8
**VDJ, CITE & DoubletFinder-training.HC_103**	702	2445	902	564	979	1102	401	14	18	253	541	116
**VDJ, CITE & DoubletFinder-training.HC_104**	1403	2213	723	534	956	837	279	38	31	242	643	143
**VDJ, CITE & DoubletFinder-training.HC_105**	2104	2930	880	703	1347	1065	475	260	54	300	635	141

Abbreviations:

VDJ-seq.*a*x (where *a* = *0.2, 0.4, 0.6, 0.8, 1.0*) refers to VDJ-identified doublets in the training set with the proportion *a* randomly subsampled.

CITE-seq.*a*x refers to CITE-identified doublets in the training set with the proportion *a* randomly subsampled.
VDJ&CITE-seq.ax refers to VDJ and CITE-identified doublets in the training set with the proportion *a* randomly subsampled.
VDJ, CITE & DoubletFinder-training.ax refers to VDJ, CITE-seq and DoubletFinder-identified doublets in the training set with the proportion *a* randomly subsampled.
METHOD*.Sample*X (where *X = 1, 2, 3 and METHOD* = VDJ-seq., CITE-seq, VDJ&CITE-seq, VDJ, CITE & DoubletFinder-training) refers to the identified doublets in via the corresponding method for the the training set from only healthy PBMC sample *X*.

Table S3. The number of total droplets, and identified doublets/multiplets with different methods, and predicted doublets using different doublet training sets for the NSCLC dataset. Related to Figure 4.

		B cell	connective tissue	DC	epithelia	Granulo-cyte	mono./ mac.	NK cell	plasma cell	T cell	Unknown
	**All droplets**	**2327**	**20**	**154**	**1149**	**148**	**782**	**140**	**147**	**1813**	**48**
**MLtiplet training doublets/ multiplets**	VDJ training	26	0	23	19	2	18	4	8	65	3
	DF training	9	0	0	0	1	4	7	0	43	3
	VDJ DF training	33	0	23	19	3	22	7	8	75	6
**MLtiplet predictions**	VDJ predicted	3	0	4	4	0	2	2	4	16	2
	DF predicted	7	0	1	0	1	0	3	0	19	2
	VDJ DF predicted	3	0	5	4	1	2	2	5	20	2

Supplementary figures

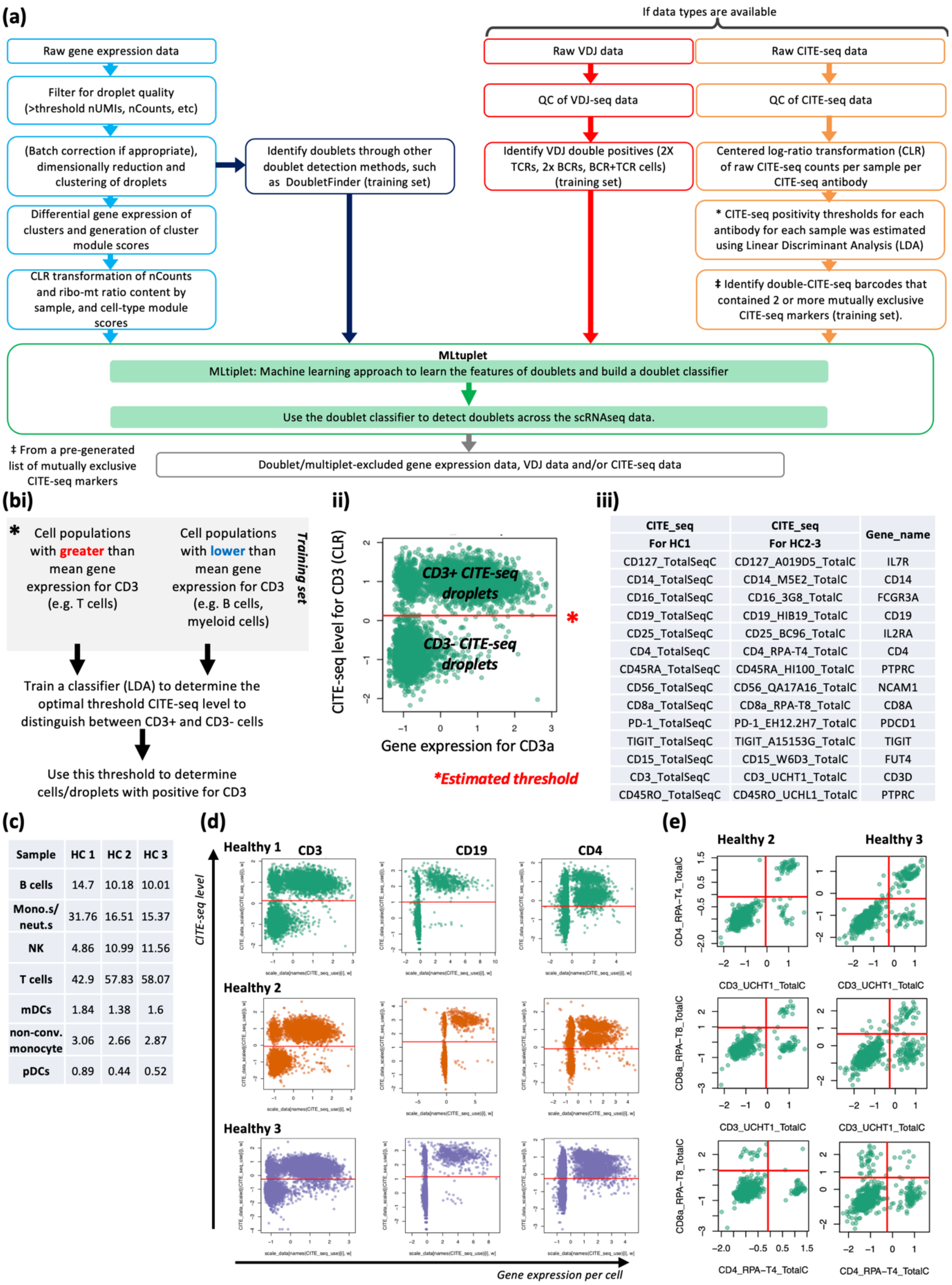

CITE_seq For HC1	CITE_seq For HC2-3	Gene_name
CD127_TotalSeqC	CD127_A019D5_TotalC	IL7R
CD14_TotalSeqC	CD14_M5E2_TotalC	CD14
CD16_TotalSeqC	CD16_3G8_TotalC	FCGR3A
CD19_TotalSeqC	CD19_HIB19_TotalC	CD19
CD25_TotalSeqC	CD25_BC96_TotalC	IL2RA
CD4_TotalSeqC	CD4_RPA-T4_TotalC	CD4
CD45RA_TotalSeqC	CD45RA_HI100_TotalC	PTPRC
CD56_TotalSeqC	CD56_QA17A16_TotalC	NCAM1
CD8a_TotalSeqC	CD8a_RPA-T8_TotalC	CD8A
PD-1_TotalSeqC	PD-1_EH12.2H7_TotalC	PDCD1
TIGIT_TotalSeqC	TIGIT_A15153G_TotalC	TIGIT
CD15_TotalSeqC	CD15_W6D3_TotalC	FUT4
CD3_TotalSeqC	CD3_UCHT1_TotalC	CD3D
CD45RO_TotalSeqC	CD45RO_UCHL1_TotalC	PTPRC

Sample	HC 1	HC 2	HC 3
B cells	14.7	10.18	10.01
Mono.s/ neut.s	31.76	16.51	15.37
NK	4.86	10.99	11.56
T cells	42.9	57.83	58.07
mDCs	1.84	1.38	1.6
non-conv. monocyte	3.06	2.66	2.87
pDCs	0.89	0.44	0.52

Figure S1. Workflow of MLtiplet doublet/multiplet detection using VDJ-seq and CITE-seq modalities. Related to Figure 1.

(a) Schematic of MLtiplet doublet detection workflow. (bi) Detailed explanation of step CITE-seq positivity thresholds for each antibody for each sample (marked by a * in (a)) using CD3 as an example. For each CITE-seq antibody, the normalised CITE-seq levels between cell populations with high corresponding gene expression (such as T cells for CD3, bii) and low gene expression (such as B cells and myeloid cells) were the input into a linear classifier to determine the optimal threshold for distinguishing the CD3 CITE-seq positive and CD3 CITE-seq negative cells/droplets. Each cell/droplet is then classified to determine whether they are positive (CD3 CITE-seq level greater than threshold) or negative (CD3 CITE-seq level lower than threshold). This is performed for each CITE-seq antibody. (biii) The available CITE-seq probes from the peripheral blood mononuclear cells (PBMCs) from three healthy individuals (https://support.10xgenomics.com/single-cell-vdj/datasets). (c) The percentages of each broad immune cell type per sample annotated through differential gene expression and CITE-seq marker expression. (d) Examples of the CITE-seq thresholds set per patient for CITE-seq antibodies targeting CD3, CD19 and CD4. (e) Examples of the CITE-seq levels between CD3, CD4 and CD8 for the healthy individuals 2 and 3 for the B cell cluster, with the red lines corresponding to the CITE-seq positivity thresholds. The equivalent plot for healthy individual 1 is provided in Figure 1.

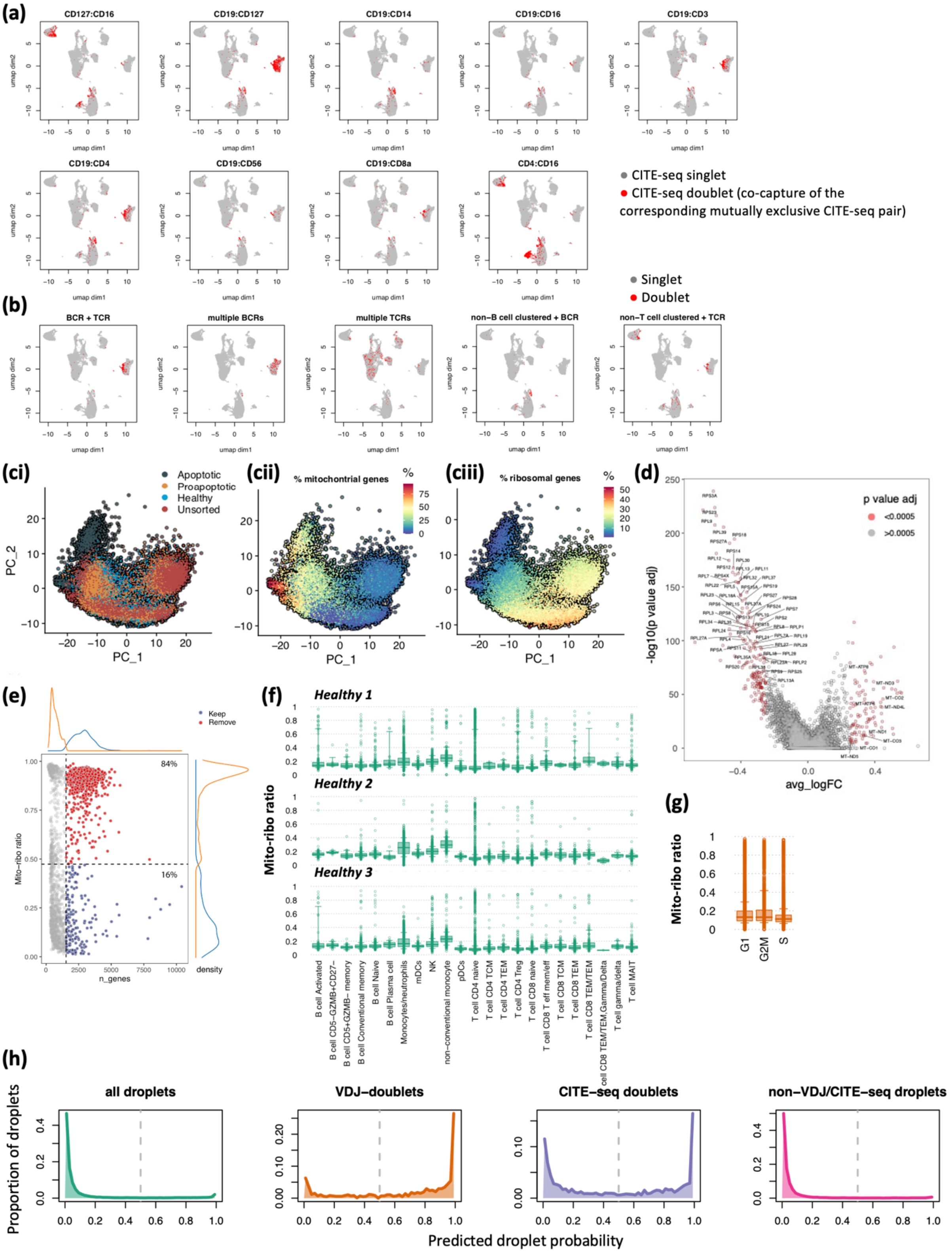
(a)
CD127:CD16
CD19:CD127
CD19:CD14
CD19:CD16
CD19:CD3
CD19:CD4
CD19:CD56
CD19:CD8a
CD4:CD16
umap dim1
umap dim2
CITE-seq singlet
CITE-seq doublet (co-capture of the corresponding mutually exclusive CITE-seq pair)
(b)
Singlet
Doublet
BCR + TCR
multiple BCRs
multiple TCRs
non−B cell clustered + BCR
non−T cell clustered + TCR
(ci)
Apoptotic
Proapoptotic
Healthy
Unsorted
PC_1
PC_2
(cii)
% mitochontrial genes
(ciii)
% ribosomal genes
(d)
p value adj
<0.0005
>0.0005
-log10(p value adj)
avg_logFC
(e)
Keep
Remove
84%
16%
Mito−ribo ratio
n_genes
density
(f)
Healthy 1
Healthy 2
Healthy 3
Mito-ribo ratio
B cell Activated
B cell CD5−GZMB+CD27−
B cell CD5+GZMB− memory
B cell Conventional memory
B cell Naive
B cell Plasma cell
Monocytes/neutrophils
mDCs
NK
non−conventional monocyte
pDCs
T cell CD4 naive
T cell CD4 TCM
T cell CD4 TEM
T cell CD4 Treg
T cell CD8 naive
T cell CD8 T eff mem/eff
T cell CD8 TCM
T cell CD8 TEM
T cell CD8 TEM/TEM
T cell CD8 TEM/TEM,Gamma/Delta
T cell gamma/delta
T cell MAIT
(g)
Mito-ribo ratio
G1
G2M
S
(h)
all droplets
VDJ−doublets
CITE−seq doublets
non−VDJ/CITE−seq droplets
Proportion of droplets
Predicted droplet probability

Figure S2. Doublet/multiplet detection using VDJ-seq and CITE-seq modalities in human healthy PBMCs and MLtiplet training features.
Related to Figure 1.
(a) The UMAP distributions of the CITE-seq doublet droplets (red) and remainder droplets (grey). (b) The UMAP distributions of the BCR/TCR doublet droplets (red) and remainder droplets (grey). (ci) PCA plot of HEK293 cells enriched for apoptotic, proapoptotic, healthy and unsorted populations. (cii) PCA coloured by percentage mitochondrial gene counts of total gene counts. (ciii) PCA coloured by percentage ribosomal gene counts of total gene counts. (d) Differential gene expression analysis between apoptotic and healthy single cells. (e) Mito-ribo ratio vs. total number of genes detected with corresponding density plots. Inferred doublets assigned to be removed are highlighted in red. Percentages shown are calculated on all cells above the local minimum of a GMM fitted on total genes detected, represented by the dotted vertical line. The mito-ribo ratios for each droplet across (f) cell types and (g) cell cycle phases per healthy PBMC sample (as defined by gene expression). (h) Histogram of doublet prediction probabilities by MLtiplet for (from left to right) all droplets, doublets/multiplets identified by VDJ-seq, doublets/multiplets identified by CITE-seq, and droplets that were not identified as doublets/multiplets identified by VDJ-seq or CITE-seq.

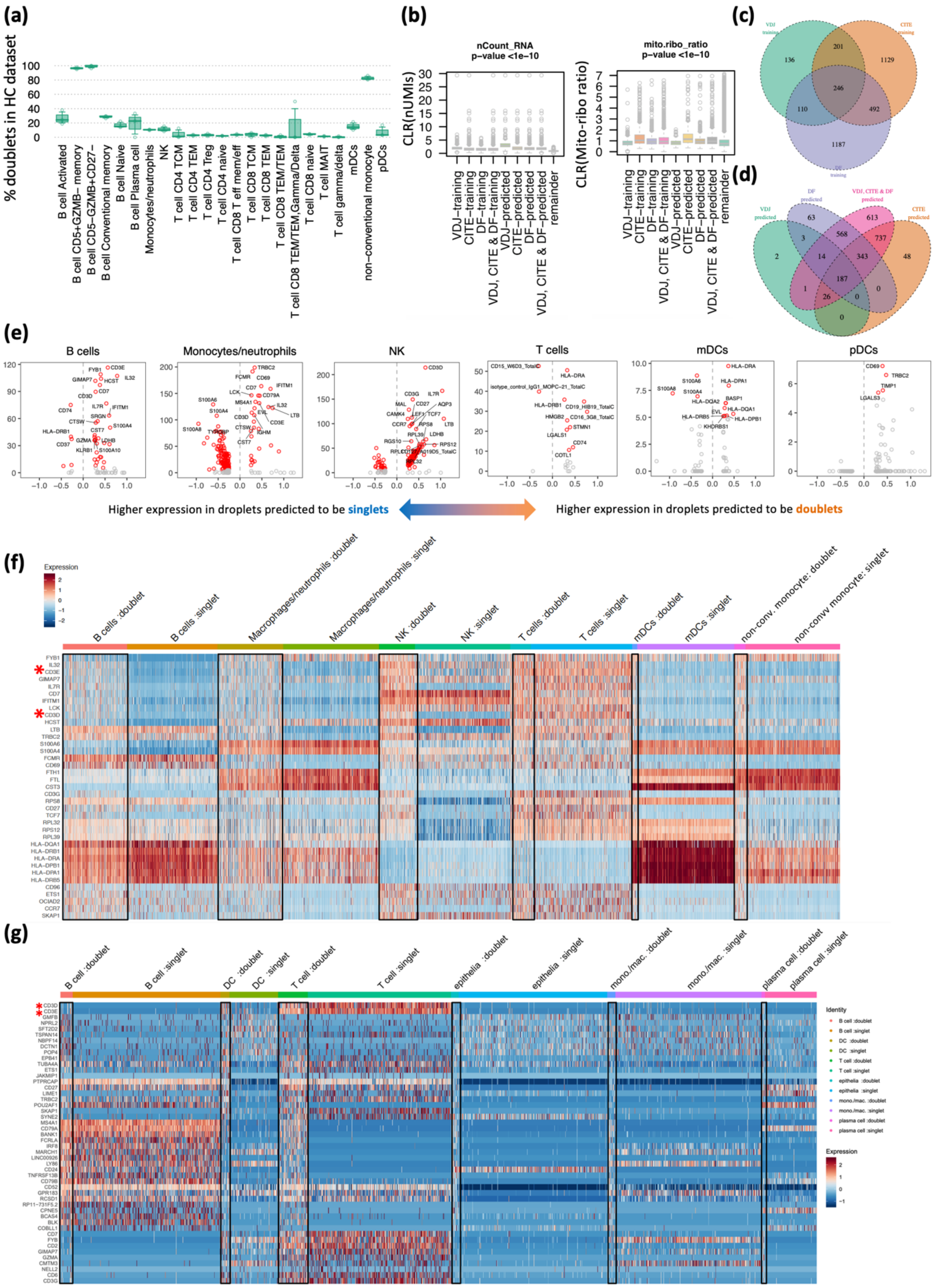

(a)
% doublets in HC dataset
B cell Activated
B cell CD5+GZMB− memory
B cell CD5−GZMB+CD27−
B cell Conventional memory
B cell Naive
B cell Plasma cell
Monocytes/neutrophils
NK
T cell CD4 TCM
T cell CD4 TEM
T cell CD4 Treg
T cell CD4 naive
T cell CD8 T eff mem/eff
T cell CD8 TCM
T cell CD8 TEM
T cell CD8 TEM/TEM
T cell CD8 TEM/TEM,Gamma/Delta
T cell CD8 naive
T cell MAIT
T cell gamma/delta
mDCs
non-conventional monocyte
pDCs
(b)
nCount_RNA
p-value <1e−10
CLR(nUMIs)
mito.ribo_ratio
p-value <1e−10
CLR(Mito-ribo ratio)
VDJ−training
CITE−training
DF−training
VDJ, CITE & DF−training
VDJ−predicted
CITE−predicted
DF−predicted
VDJ, CITE & DF−predicted
remainder
(c)
VDJ training
CITE training
DF training
(d)
VDJ predicted
DF predicted
VDJ, CITE & DF predicted
CITE predicted
(e)
B cells
Monocytes/neutrophils
NK
T cells
mDCs
pDCs
Higher expression in droplets predicted to be singlets
Higher expression in droplets predicted to be doublets
(f)
Expression
B cells :doublet
B cells :singlet
Macrophages/neutrophils :doublet
Macrophages/neutrophils :singlet
NK :doublet
NK :singlet
T cells :doublet
T cells :singlet
mDCs :doublet
mDCs :singlet
non-conv. monocyte: doublet
non-conv monocyte: singlet
(g)
B cell :doublet
B cell :singlet
DC :doublet
DC :singlet
T cell :doublet
T cell :singlet
epithelia :doublet
epithelia :singlet
mono./mac. :doublet
mono./mac. :singlet
plasma cell :doublet
plasma cell :singlet
Identity
Expression

Figure S3. Features of training and predicted doublet/multiplet sets.
Related to Figure 2.

(a) Percentages of predicted doublets by MLtiplet per cell annotation in healthy scRNA-seq dataset. (b) The relative numbers of RNA molecules (nUMI) and mito-ribo ratio (mitoribo_ratio) per droplet for the VDJ-identified doublets/multiplets, the CITE-seq-identified and DoubletFinder (DF)-identified doublets/multiplets (training) and the resulting predicted doublets/multiplets derived from these training datasets using MLtuplet. "Remainder" refers to droplets that were not identified/predicted to be doublets/multiplets from the VDJ-seq or CITE-seq data. The p-values of the differences between the feature distributions of the doublet/multiplets detected and the remainder of the droplets provided (two-sided Wilcoxon test). Venn diagrams of the numbers of droplets in (c) each training set by method, and (d) predicted from MLtuplet using each training set. (e) Volcano plots of the differential gene expression between droplets predicted to be doublets/multiplets compared to those predicted to be singlets per UMAP cluster. The top 20 genes with a p-value <1e5 are labelled. (f) Heatmap of differential gene expression between healthy PBMC between predicted singlets and doublets/multiplets per cell type cluster. (g) Heatmap of differential gene expression between predicted singlets and doublets/multiplets per cell type cluster.

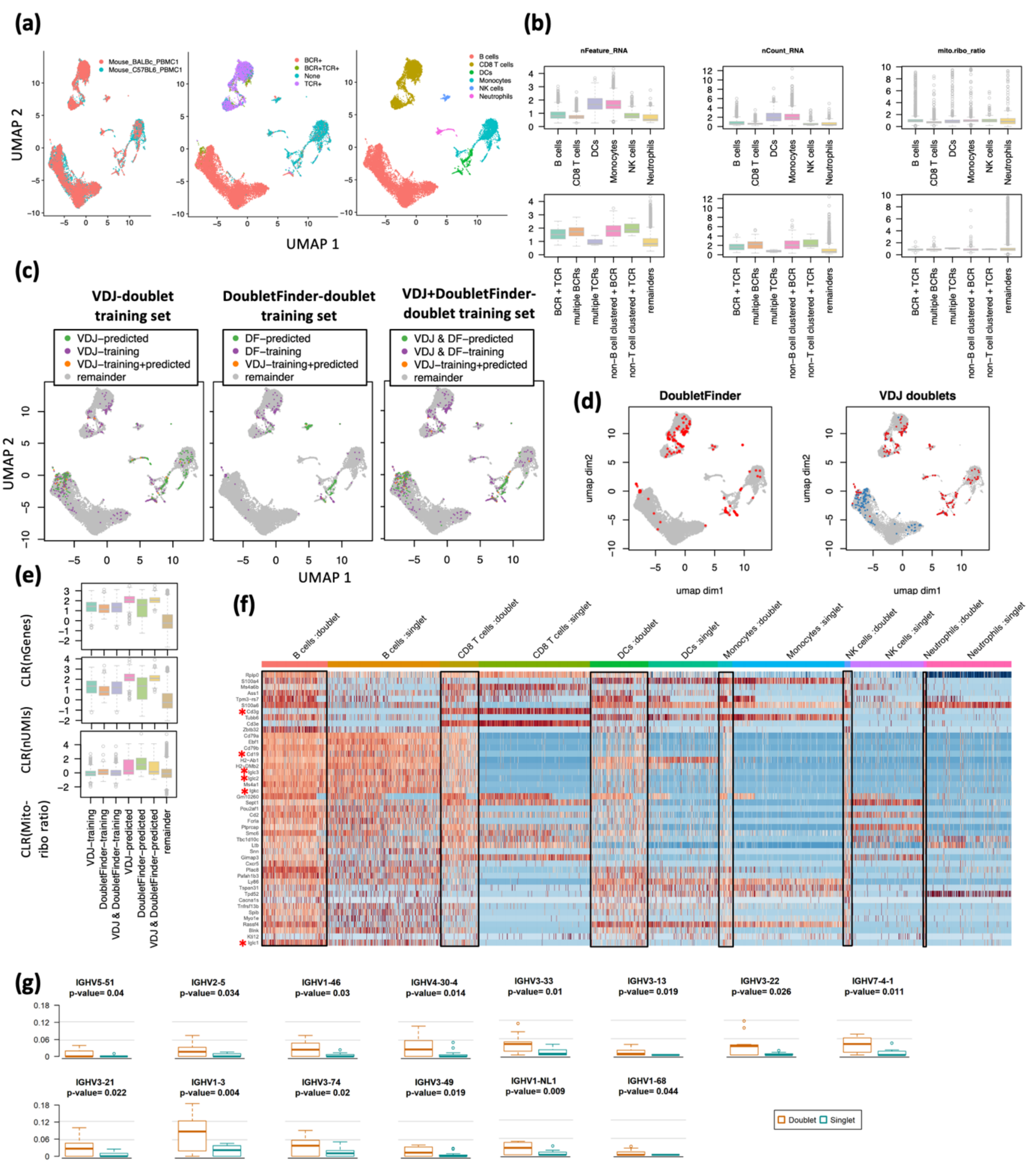

Figure S4. MLtiplet training features and predictions from a murine PBMC dataset. Related to Figure 4.

Doublet detection on a murine dataset comprising PBMCs from two mouse strains (BALB/c and C57BL/6. (a) UMAP plots of (left) each mouse sample, (middle) VDJ-seq information, and (right) the annotated cell types. (b) The relative numbers of genes (nFeatures), RNA molecules (nUMI) and mito-ribo ratio (mitoribo_ratio) per cell for (top) each cell type, and (bottom) the VDJ-identified doublets/multiplets. (c) UMAP plot of the doublets identified from (left) DoubletFinder and (right) VDJ-seq heterotypic doublets. (d) The relative numbers of genes (nFeatures), RNA molecules (nUMI) and mito-ribo ratio (mitoribo_ratio) per cell for the VDJ-identified doublets/multiplets, DoubletFinder-identified doublets/multiplets, MLtiplet predicted doublets/multiplets and the remainder (predicted singlets by MLtiplet). (e) UMAP plots of the training and predicted doublets/multiples using each approach. (f) Heatmap of differential gene expression between murine PBMC between predicted singlets and doublets/multiplets per cell type cluster. (g) Enrichment of IGHV gene usages between doublets and singlets in three healthy peripheral blood samples. Tests performed by MANOVA in R.
